# Yin Yang Gene Expression Ratio Signature for Lung Cancer Prognosis

**DOI:** 10.1371/journal.pone.0068742

**Published:** 2013-07-17

**Authors:** Wayne Xu, Shantanu Banerji, James R. Davie, Fekadu Kassie, Douglas Yee, Robert Kratzke

**Affiliations:** 1 Manitoba Institute of Cell Biology, University of Manitoba, Winnipeg, Canada; 2 Manitoba Institute of Child Health, University of Manitoba, Winnipeg, Canada; 3 Faculty of Pharmacy, University of Manitoba, Winnipeg, Canada; 4 Section of Hematology and Oncology, Department of Internal Medicine, University of Manitoba, Winnipeg, Canada; 5 Department of Biochemistry and Medical Genetics, University of Manitoba, Winnipeg, Canada; 6 Department of Medical Oncology and Hematology, CancerCare Manitoba, Winnipeg, Canada; 7 Masonic Cancer Center, University of Minnesota, Minneapolis, Minnesota, United States of America; 8 Division of Hematology, Oncology and Transplantation, Department of Medicine, University of Minnesota, Minneapolis, Minnesota, United States of America; The Norwegian University of Science and Technology (NTNU), Norway

## Abstract

Many studies have established gene expression-based prognostic signatures for lung cancer. All of these signatures were built from training data sets by learning the correlation of gene expression with the patients' survival time. They require all new sample data to be normalized to the training data, ultimately resulting in common problems of low reproducibility and impracticality. To overcome these problems, we propose a new signature model which does not involve data training. We hypothesize that the imbalance of two opposing effects in lung cancer cells, represented by Yin and Yang genes, determines a patient’s prognosis. We selected the Yin and Yang genes by comparing expression data from normal lung and lung cancer tissue samples using both unsupervised clustering and pathways analyses. We calculated the Yin and Yang gene expression mean ratio (YMR) as patient risk scores. Thirty-one Yin and thirty-two Yang genes were identified and selected for the signature development. In normal lung tissues, the YMR is less than 1.0; in lung cancer cases, the YMR is greater than 1.0. The YMR was tested for lung cancer prognosis prediction in four independent data sets and it significantly stratified patients into high- and low-risk survival groups (p = 0.02, HR = 2.72; p = 0.01, HR = 2.70; p = 0.007, HR = 2.73; p = 0.005, HR = 2.63). It also showed prediction of the chemotherapy outcomes for stage II & III. In multivariate analysis, the YMR risk factor was more successful at predicting clinical outcomes than other commonly used clinical factors, with the exception of tumor stage. The YMR can be measured in an individual patient in the clinic independent of gene expression platform. This study provided a novel insight into the biology of lung cancer and shed light on the clinical applicability.

## Introduction

Lung cancer is the leading cause of cancer-related deaths in North America. While there has been a decrease in lung cancer deaths among men due to a reduction in tobacco use over the past 50 years, it still accounts for 29% of all male cancer deaths in 2010 [1]. The 5-year overall survival rate for lung cancer is as low as 16% and has not significantly improved over the past 30 years [1]. Non-small cell lung cancer (NSCLC) is the most commonly diagnosed lung cancer accounting for 85% of annual cases. About 25% to 30% of NSCLC patients present with early stage I disease and receive surgical intervention. However, more than 20% of these patients relapse within five years [2]. Adjuvant therapy has improved survival of a subset of patients with stage II and III disease. However, it is not known which patients are more likely to relapse and would benefit more from additional therapies.

To improve clinical outcomes, researchers have invested much effort into identifying lung cancer biomarkers which allow clinicians to make an early diagnosis, predict disease course, and effect of treatment. Genome-wide expression profiling using microarray techniques has identified potential gene signatures to classify patients into different survival outcome cohorts [3]–[17]. Previously reported models were built by learning the correlation coefficients between gene expression and patients’ survival time from training data sets and they require that new test data sets be normalized to the training data. Consequently, these signatures have low reproducibility and are impractical in a clinic setting. There is little evidence that any of the reported gene expression signatures are ready for clinical application [18].

To address these problems, we developed an empirical model which is not based on the knowledge of patients' survival time for determining the lung cancer biomarker signature. Gene regulation is a complex multidimensional process which includes a spectrum of genes that are either activated or suppressed, and whose expression is either continuous or temporary. We hypothesize that the prognosis is determined by two opposing groups of genes which we term Yin and Yang. In lung cancer cells, the normal gene expression is dysregulated resulting in cellular proliferation and diminished differentiation. The power of Yin Yang theory is that it simplifies complex multi-dimensional aspects of gene expression into two opposing dimensions - Yin and Yang, and where the balance between Yin and Yang ensures a healthy status for cells. Previously published studies have referred to the opposing functions of known tumor suppressors and oncoproteins as yin and yang in tumorigenesis [19]–[21]. We hypothesize that instead of an individual gene, two functionally imbalanced groups of genes (Yin and Yang) in lung cancer cells determine the fate of the tumor cells, which ultimately determines patient’s survival time. Accurate identification of the Yin and Yang genes in tumor development can be used to develop a prognostic signature.

## Materials and Methods

### Lung Cancer Patient Sample Data

We focused our study on adenocarcinoma as it is a more common lung cancer and the gene expression data with associated clinical information is more readily available. The sample data from Bhattacharjee *et al*. has been described previously [22]. It consists of 203 lung cancer patient samples including 139 adenocarcinomas, 20 pulmonary carcinoids, 21 squamous cell carcinomas, 6 small-cell lung cancers, and 17 normal lung tissue samples from adjacent sections. Among the 139 adenocarcinomas, 125 patient samples were associated with clinical follow up information of survival time and recurrence. The sample data from Bild *et al*. contains 58 primary adenocarcinomas collected through the Duke Lung Cancer Prognostic Laboratory [23]. These samples were associated with 1–6 years of patients’ follow up. The National Cancer Institute Director’s Challenge Consortium (DCC) for the Molecular Classification of Lung Adenocarcinoma samples consists of 442 adenocarcinomas with patients’ clinical information [13]. These samples were collected and processed in 4 independent Institutions: Canada/Dana-Farber Cancer Institute (CAN/DF), University of Michigan Cancer Center (UM), H.L. Moffitt Cancer Center (HLM), and Memorial Sloan-Kettering Cancer Center (MSK). Stages I, II and III adenocarcinomas were collected, with approximately 60% of samples from stage I tumors. None of the patients received preoperative chemotherapy or radiation and at least 2 years of follow-up information was available. 288 lung adenocarcinoma (LUAD) samples from The Cancer Genome Atlas (TCGA) Project have comprehensive clinical information. Excluded from the dataset were 20 patients whose survival time is not available and 9 living patients with follow up time less than 2 days.

### Gene Expression Data

The gene expression of the Bhattacharjee samples was detected by Affymetrix HU_U95Av2 GeneChip. The raw hybridization intensity data files (CEL) were downloaded from http://www.broadinstitute.org/mpr/lung/. The gene expression indices were processed with the MAS5.0 algorithm by using the Expressionist Refiner module (GeneData, Inc, San Francisco, CA, USA). No further normalization was done within each data set in order to keep the individual sample independent in gene biomarker detection. Except in clustering analysis for differentially expressed gene identifications, the Robust Multi-array Average (RMA) derived and normalized expression measurements were calculated from the raw CEL files. The gene expression of the Bild samples was detected by Affymetrix HU_U133plus2 GeneChip and the signal intensity was calculated by MAS5.0 algorithm. The data set was downloaded from NCBI GEO database (GSE3141). The DCC raw HG_U133A CEL files were downloaded from NCI caArray database (https://array.nci.nih.gov/caarray/project/details.action?project.id=182) [13]. MAS5.0 algorithm was used for gene expression summarization. No normalization or prefiltering was applied to samples or genes. The 259 RNA-seq data were downloaded from TCGA Data Portal (http://tcga-data.nci.nih.gov/tcga/tcgaDownload.jsp). The gene expression RKPM (reads per kilobase per million mapped reads) value was extracted from sample files.

### Signature Genes Identification and Selection

The expression indices were summarized by RMA algorithm and further normalized by itemwise Z-normalization using Genedata Analyst module (GeneData, Inc, San Francisco, CA, USA). 2-D hierarchical Euclidean L2 distance clustering with complete linkage setting for both genes and samples was performed to explore the differentially expressed biomarker genes in lung tumors. Unregulated and downregulated genes in cancer tissues were selected from 2D clustering. Genes that were expressed higher in normal lung tissues than in lung cancer cells were called “Yang” gene candidates, conversely genes expressed higher in lung cancer cells than normal lung tissues were called “Yin” gene candidates. These two gene lists were inputted into IPA9.0 (Ingenuity® Systems, www.ingenuity.com) for interaction network and pathway analysis. The networks are built by direct interactions. The networks with significant scores were selected for further analysis.

### Gene Signature Classifier Development

The expression values of the selected Yin genes and Yang genes were extracted from published microarray expression data. Initially, the Yin (Y) and Yang (y) expression arithmetic mean ratio (YMR) was calculated as a signature classifier for each sample (YMR = 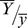). Since 31 Yin genes and 32 Yang genes were identified as probe sets from HG-U95A GeneChip, we used these probe sets to extract the Yin and Yang gene expression values of Bhattacharjeès samples. To extract Yin and Yang genes from different platforms, we used these 63 probe sets and/or their gene symbols to match the probe sets of other platforms. We first looked at the best match probe sets which share high sequence identity and represent the same genes. The best match probe set files can be downloaded from Affymetrix (http://www.affymetrix.com ). If the best match probe sets cannot be found in a particular platform, we used the Yin and Yang gene symbols. One Yin or Yang gene symbol may contain a single probe set (single match) or several probe sets. For multiple IDs within the same gene symbol, an average value was used. In HG-133plus2 of Bild data set, 62 genes have been computed for average expression values from multiple probe sets since only one best matched probe set to HG-U95A 39651_at (RECQL4 gene). In HG-133A platform of DCC data set, 22 Yin genes’ expression was derived from 22 best matched probe sets, 3 genes match single probe sets and 6 genes’ expression was averaging expression of multiple probe sets; 29 Yang genes’ expression was from best matched probe sets, and 2 genes from multiple probe sets. The patient risk score was derived from the YMR values. Using a YMR cutoff values, we divided patients into high- and low-risk prognostic groups. Since a 2-fold difference is often chosen as an arbitrary value in a two-group comparison, we defined a 2-fold Yin over Yang as a cutoff and then adjusted it based on normal sample mean YMR or cancer sample mean YMR. If the normal lung sample YMR is significantly less than 1.0 (for example, the TCGA RNAseq data), the YMR cutoff will be adjusted to be lower than 2.0. If normal sample mean YMR is not available for a particular data set (for example, DCC and Bild data sets), we adjusted a cutoff value that is close to the mean YMR of the lung cancer data set since many studies use the mean risk score to stratify patients. The expression value of a gene may be measured from a single probe set in one platform but multiple probe sets in other platform. This difference in expression measurement could result in different YMR cutoff values in different platforms. We expect the same YMR cutoff value for the same platform. It is worth to note that these large scale expression platforms were originally designed for research purpose, not for clinical use. The arbitrary YMR cutoff values determined from these different platforms are only used for the YMR signature validation. In future, we will optimize a single YMR cutoff value for results from a clinically relevant platform such as qPCR.

We also compared the arithmetic YMR with geometric Mean of Yin and Yang Ratio (gYMR). To test optimal gene size, we observed the effect of dropping genes from the 31 Yin and 32 Yang gene list on the association with clinical outcome. We also assessed the significance of the YMR signature by comparing YMR to ratio of randomly picked groups of identical group size.

### Statistical Analysis

To evaluate the performance of the YMR signature, we used each YMR as dichotomous or continuous covariate in a Cox proportional hazards model, with 5–6 years overall survival or recurrence-free as the outcome variable [13], [24]–[26]. The estimated hazard ratio, 95% confidence interval and p-value allowed us to directly compare the performances of YMR covariate with other clinical variables. Kaplan-Meier product-limit methods and log-rank tests were used to estimate and test differences in probability of survival between low- and high-risk patient groups. The survivor function was plotted for each subgroup. All statistical analyses were performed using Partek® software, version 6.3 (Partek Inc., St. Louis, MO, USA) or R statistic package Survcomp [27].

### Validation

In order to validate that the YMR is less than 1.0 in normal lung tissues and greater than 1.0 in lung cancer tissue samples, we measured the YMR in new independent data sets. These data sets were processed by different platforms including Affymetrix GeneChip HG-U95, HG-133A, HG-133plus2, Illumina beadChip, and two-channel array. The YMRs were calculated from these data sets either with or without data normalization based on the original data sources.

To validate the YMR signature for lung cancer prognosis, four independent data sets were used: 125 Bhattacharjee adenocarcinomas sample data set of HG_U95Av2 platform of which the survival time was not used in the model building, 58 Bild adenocarcinomas sample data of HG-133Plus2 platform, 442 DCC sample files of HG-133A platform, and 259 TCGA samples of RNA-seq platform. These are well-defined patient samples with clinical information. For analyses in this study, survival or recurrence-free outcomes were compared according to high-risk YMR (i.e. YMR is greater than 2.0 or an adjusted cutoff) and low-risk YMR (YMR is less than or equal to 2.0 or an adjusted cutoff) patients. The YMR score stratification in the same stages and in response to treatment was tested in the following groups of the DCC patients, respectively: stage I; stage II & III; received chemotherapy; no chemotherapy; chemotherapy on stage I; chemotherapy on stage II & III; no chemotherapy on stage I; no chemotherapy on stage II & III.

## Results

### Identification of Candidate Lung Cancer Biomarker Genes

**Figure 1:**
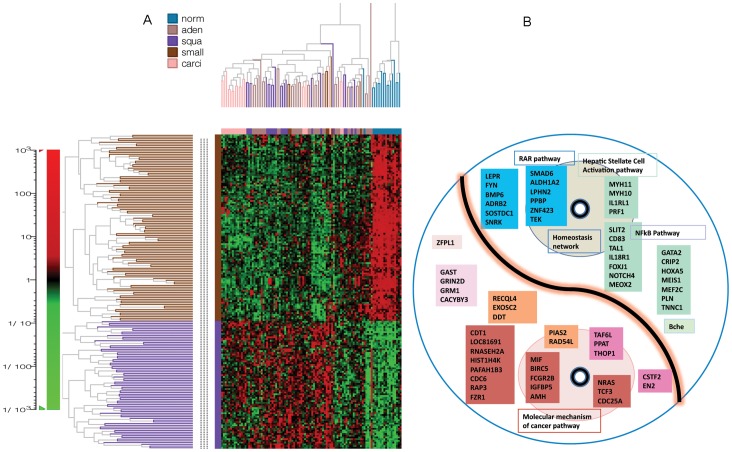
Identification and selection of Yin and Yang genes. **A**. Clustering of gene identification. The probe sets are in rows and the samples are in columns. The expression indexes of all the 12,625 probe sets of the 100 samples were summarized by RMA algorithm and further normalized by itemwise Z-normalization. 74 upregulated genes (bottom half rows) and 108 (top half rows) down regulated genes in cancer tissues were selected from the 2D clustering regions. The preselected 74 and 108 probsets were displayed by clustering again. **B**. Yin (bottom) and Yang (top) genes selection by functional analysis. The two circles represent the two cores of functional effects of the Yin and the Yang. The genes highlighted by the same color are in the same interaction network.

We compared normal lung samples with the lung cancer samples collected from patients of mixed tumour stages with different survival times to identify and select genes groups for signature development. Using unsupervised clustering analysis of microarray data from Bhattacharjee *et al*. [22], we examined the differential gene expression in 17 normal lung tissue samples and 83 samples from a variety of lung cancer types. In the 2D clustering, we selected a region where the genes downregulated in normal samples but upregulated in almost all types of lung cancers (Figure S1A). The region where genes were upregulated in one or a few cancer types was not selected. We identified 74 probe sets in this region (Figure S1B, Table S1). We also identified a region where genes were upregulated in normal samples but downregulated in almost all types of lung cancers (Figure S2A). The region where genes were downregulated in one or few cancer types was not selected. We identified 108 probe sets in this region (Figure S2B, Table S2, Figure 1A).

By comparing gene expression between various cell types of lung cancer to the normal lung cells, common Yin and Yang genes among the different cancers could be identified. Gene clustering, rather than group statistic test, not only detects the expression patterns, but also indicates some extent of the gene interactions within the same pattern. Unlike differential gene expression resulting from two-group statistic tests, gene expression patterns resulting from clustering has greater tolerance to variations due to sample collection and data processing. Individual genes may not present in the differential gene list due to large variations found in a few samples, but the same genes may show a similar overall expression pattern in cluster analysis.

Yin Yang genes showed little overlap with the previously reported lung cancer prognostic signature genes. However, many Yin genes reported here were found in previous studies that relate lung cancer or other tissue type cancer development such as GRIN2D [28], GAST [29], AMH [30], TCF3 [31], EXOSC2 [32], GRM1 [33], CDT1 [34], RecQL4 [35], CSTF2 [36], FCGR2B [37], RNASEH2A [38], CDC6 [39], CACYBP [40], BIRC5 [41], CDC25 [42], NRAS [43], EN2 [44], and MIF [45]. Although the *n-ras* proto-oncogene is in the Yin gene list, we did not find other oncogenes that are involved in lung tumorigenesis. This may be due to alteration of different oncogenes in different subsets of lung cancers. However, we speculate that progression genes may play more important role than genes involved in the initiation or promotion stage of lung tumorigenesis in determining the lung cancer prognosis.

Pathway and interaction network analyses of these 74 genes allowed selecting two main networks that are related to tumor morphology (Table S3, network significant score of 42) and DNA replication (Table S4, network significant score of 30). These networks participate in the canonical Molecular Mechanisms of Cancer pathway (Figure 1B, Figure S3). These networks contain 31 genes whose gene symbol names matched the Affymetrix U95 AV2 probe set identifiers. We selected these 31 genes as Yin gene candidates (Table 1). The 108 downregulated genes constituted two main networks related to maintenance (network significant score of 63) and cellular development (network significant score of 23) processes. The RAR Activation pathway and the Hepatic Stellate Cell Activation pathway (Figure S4) invoked by Yang genes exert a wide variety of effects on tissue homeostasis, cell proliferation, differentiation, and apoptosis. There is evidence that lung tissue harbors Hepatic Stellate-like cells which are vitamin-A-storing lung cells [46]–[47]. We retrieved the focus genes from the networks that involved cell maintenance and cellular development process resulting in two gene groups. These two groups (Table S5, S6) were combined, resulting in 32 unique genes in total. We defined these 32 genes as Yang gene candidates for signature development (Table 2).

**Table 1:** Yin genes.

HG_U95A probe set	Gene Symbol	Gene Title
31711_at	GRIN2D	glutamate receptor, ionotropic, N-methyl D-aspartate 2D
34552_at	GAST	gastrin
35084_at	AMH	anti-Mullerian hormone
32874_at	TCF3	transcription factor 3 (E2A immunoglobulin enhancer binding factors E12/E47)
32975_g_at	EXOSC2	exosome component 2
33510_s_at	GRM1	glutamate receptor, metabotropic 1
34027_f_at	HIST1H4J///HIST1H4K	histone cluster 1, H4j///histone cluster 1, H4k
34510_at	CDT1	chromatin licensing and DNA replication factor 1
37432_g_at	PIAS2	protein inhibitor of activated STAT, 2
39651_at	RECQL4	RecQ protein-like 4
40334_at	CSTF2	cleavage stimulation factor, 3' pre-RNA, subunit 2, 64 kDa
41623_s_at	FZR1	fizzy/cell division cycle 20 related 1 (Drosophila)
34664_at	FCGR2B	Fc fragment of IgG, low affinity IIb, receptor (CD32)
35141_at	RNASEH2A	ribonuclease H2, subunit A
36839_at	CDC6	cell division cycle 6 homolog (S. cerevisiae)
37267_at	THOP1	thimet oligopeptidase 1
41149_at	LOC81691	exonuclease NEF-sp
33935_at	CACYBP	calcyclin binding protein
34341_at	PPAT	phosphoribosyl pyrophosphate amidotransferase
35800_at	PAFAH1B3	platelet-activating factor acetylhydrolase 1b, catalytic subunit 3 (29 kDa)
39909_g_at	TAF6L	TAF6-like RNA polymerase II, p300/CBP-associated factor (PCAF)-associated factor, 65 kDa
40264_g_at	ZFPL1	zinc finger protein-like 1
40532_at	BIRC5	baculoviral IAP repeat-containing 5
1738_at	CDC25A	cell division cycle 25 homolog A (S. pombe)
1601_s_at	IGFBP5	insulin-like growth factor binding protein 5
1539_at	NRAS	neuroblastoma RAS viral (v-ras) oncogene homolog
1133_at	EN2	engrailed homeobox 2
966_at	RAD54L	RAD54-like (S. cerevisiae)
895_at	MIF	macrophage migration inhibitory factor (glycosylation-inhibiting factor)
652_g_at	RPA3	replication protein A3, 14 kDa
374_f_at	DDT///DDTL	D-dopachrome tautomerase///D-dopachrome tautomerase-like

**Table 2:** Yang genes.

HG_U95A Probe set	Gene Symbol	Gene Title
34174_s_at	LPHN2	latrophilin 2
36377_at	IL18R1	interleukin 18 receptor 1
32904_at	PRF1	perforin 1 (pore forming protein)
34950_at	ZNF423	zinc finger protein 423
37841_at	BCHE	butyrylcholinesterase
39209_r_at	PPBP	pro-platelet basic protein (chemokine (C-X-C motif) ligand 7)
39577_at	SOSTDC1	sclerostin domain containing 1
39634_at	SLIT2	slit homolog 2 (Drosophila)
40322_at	IL1RL1	interleukin 1 receptor-like 1
40398_s_at	MEOX2	mesenchyme homeobox 2
41030_at	FOXJ1	forkhead box J1
34267_r_at	LEPR	leptin receptor
37194_at	GATA2	GATA binding protein 2
37536_at	CD83	CD83 molecule
38315_at	ALDH1A2	aldehyde dehydrogenase 1 family, member A2
39048_at	NOTCH4	Notch homolog 4 (Drosophila)
39085_at	TNNC1	troponin C type 1 (slow)
40763_at	MEIS1	Meis homeobox 1
35828_at	CRIP2	cysteine-rich protein 2
37710_at	MEF2C	myocyte enhancer factor 2C
38734_at	PLN	phospholamban
39544_at	SYNM	synemin, intermediate filament protein
40231_at	SMAD6	SMAD family member 6
40900_at	MYH10	myosin, heavy chain 10, non-muscle
2039_s_at	FYN	FYN oncogene related to SRC, FGR, YES
1733_at	BMP6	bone morphogenetic protein 6
1595_at	TEK	TEK tyrosine kinase, endothelial
873_at	HOXA5	homeobox A5
774_g_at	MYH11	myosin, heavy chain 11, smooth muscle
610_at	ADRB2	adrenergic, beta-2-, receptor, surface
560_s_at	TAL1	T-cell acute lymphocytic leukemia 1
481_at	SNRK	SNF related kinase

### Gene Signature for Lung Cancer

**Figure 2:**
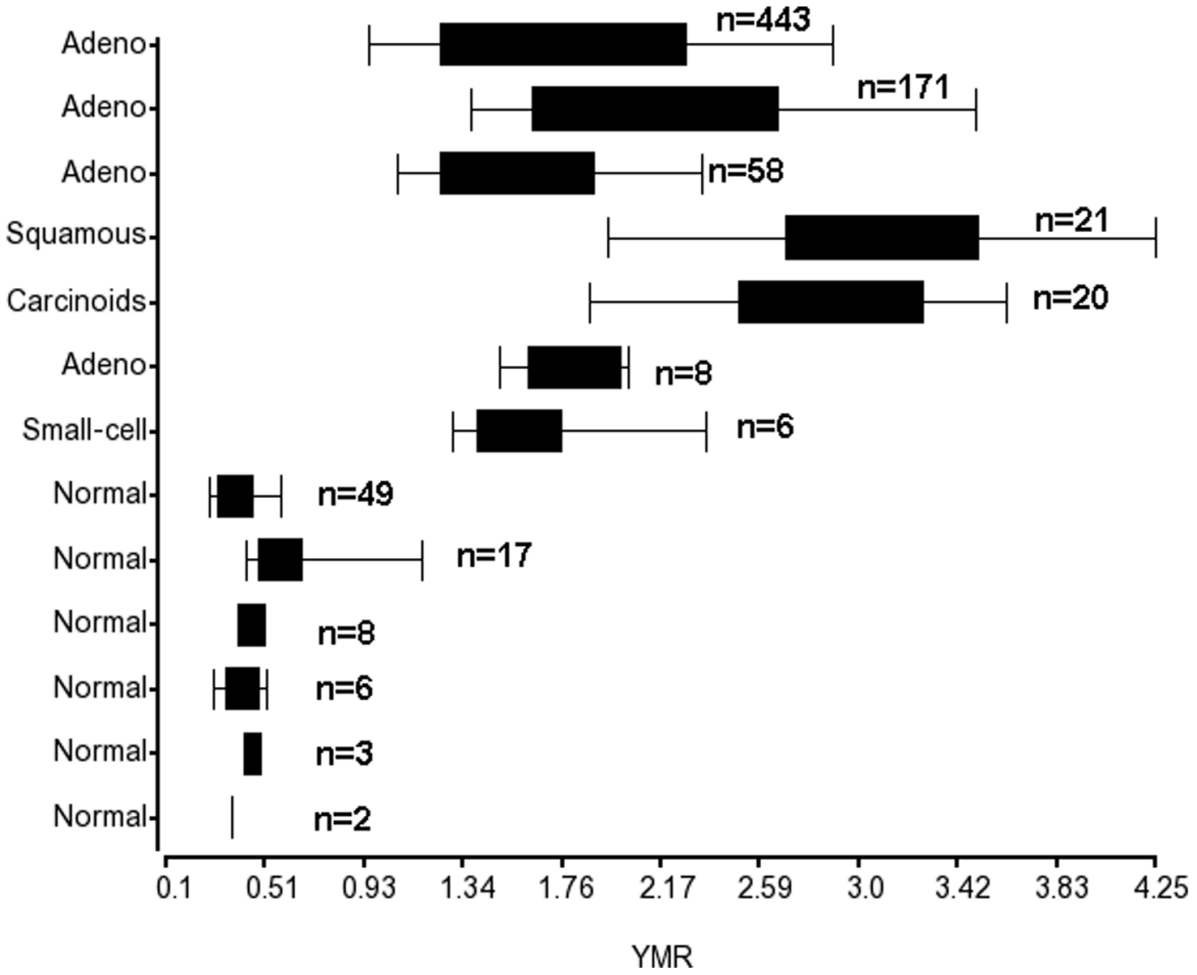
Boxplot of the YMR distributions in normal lung samples and lung cancer samples. Microarray gene expression data sets from different reports with different platforms were used. The data sets were described as in Table S7.

To build the signature model, we computed the YMR to the patient risk scores. The YMR represents a simple combination or interaction effect of the Yin genes and Yang genes. The ratio indicates the Yin and Yang balance status in lung cells or which group of genes is more active than others and the extent of this difference. In normal lung cells, the Yang is greater than Yin. Cancer phenotypes have higher YMR scores then are associated with higher risk disease. We first validated our hypothesis that YMR is less than 1.0 in normal lung tissues and greater than 1.0 in lung cancer tissues. We used several independent sample data sets with different platforms and different preprocesses (Table S7). YMRs were less than 1.0 in all normal lung data sets [48]–[52] (Figure 2). We also measured the YMRs of 12 different normal human tissue types in one data set [52] (Table S8). The YMRs were less than 1.0 in normal lung, as well as in other normal tissues such as the heart, spleen, skeletal muscle, and prostate, but greater than 1.0 in other tissues such as the liver. This result suggests that the Yin and Yang gene expression profiles are tissue type specific. In the 83 samples of various lung cancer types from which Yin and Yang genes were identified via differential gene expression analysis, all samples had an YMR greater than 1.0. The YMRs greater than 1.0 in other independent lung cancer sample data sets are also shown in Figure 2.

### YMR Signature Predicts Survival Outcomes

**Figure 3:**
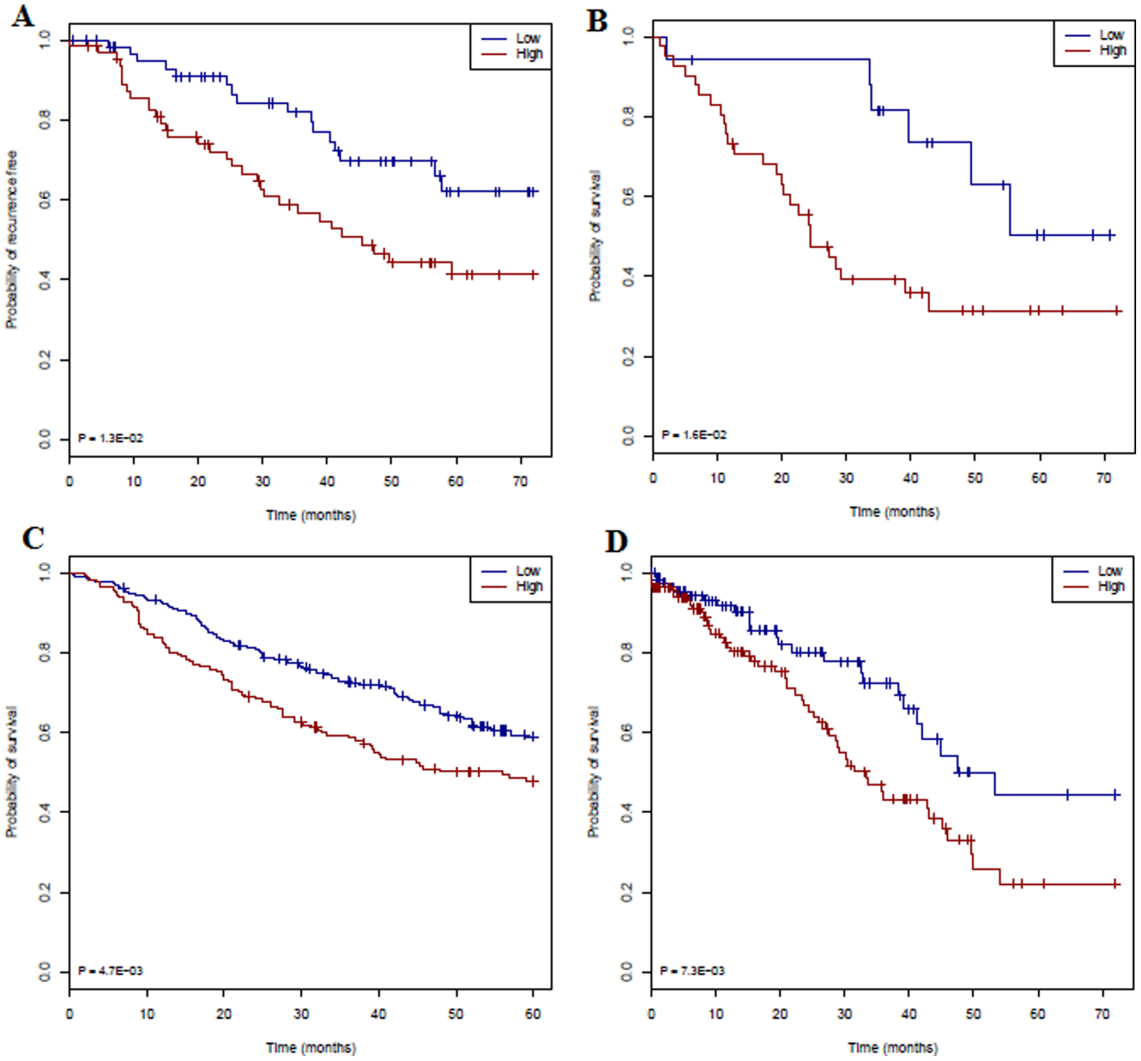
Validation of YMR in four data sets by Kaplan-Meier estimates of the survivor function. **A.** Free-recurrence time function curve (low risk n = 60; high risk n = 65) of the adenocarcinomas patients from Bhattacharjee *et al*. **B**. Overall survival time function curve of the adenocarcinomas patients (low risk n = 27; high risk n = 31) from Bild *et al*. **C**. Patient samples (low risk n = 248; high risk n = 194) of the DCC project. **D**. RNA-seq samples (low risk n = 121; high risk n = 137) from TCGA. Low YMR scores (in green) correspond to the highest predicted survival probability and high YMR scores (in red) correspond to the greatest predicted risk.

We evaluated the YMR for prognosis of four data sets in which the patient clinical information was available. We first validated the YMR model for the risk outcome of the Bhattacharjee data set [22] from which the model was built. Since the patients' survival time or recurrence-free time information was not used in the modeling, this data set therefore serves as an independent data set. We first tested the YMR as a continuous variable using proportional hazard model and proved that the increased YMR is associated with poorer outcomes within 6-year recurrence free rate (p = 0.044, HR = 1.96) (Table S9). We then examined the YMR as a dichotomous variable to stratify patients as high and low risk groups. Since the normal lung samples from the same data set shows a mean YMR of 0.91 and the 125 adenocarcinomas have a mean YMR of 2.23, we defined a YMR cutoff of 2.0. We grouped 125 adenocarcinomas patients into high risk (YMR>2.0, n = 65) and low risk (YMR< = 2.0, n = 60) groups. As seen in Figure 3A, the YMR significantly stratified the high recurrence and low recurrence risk groups (p = 0.013, HR = 2.7). Previous studies have reported a significant p-value for their gene-signatures. This is to be expected as those signatures were developed by the patients’ survival time and then used again to predict survival time. As subsequently demonstrated, the problem with these approaches is their low reproducibility for new independent data sets. By contrast, the YMR approach is not trained to a specific dataset and would be assumed to work for any data set. We randomly picked 500 pairs of groups of identical group sizes of Yin and Yang genes from 12,625 genes of the HU-95av2 platform and used the same ratio cutoff as the YMR >2.0. The 500 p-values have a mean p-value of 0.75 (sd = 0.32) (Figure S5). We did find that four p-values from these random tests are very low (0, 0, 0, 1E-18, respectively), however their HRs are 1.0 or close to 1.0 thus these groups cannot stratify risk groups.

We then evaluated the YMR for a large independent DCC data set. These data sets were collected and processed from four different institutions. They contained pathological data and clinical information describing the severity of the disease at surgery and the clinical course of the disease after sampling [13]. We grouped these 442 patients by YMR into high risk (YMR>1.8, n = 194) and low risk (YMR< = 1.8, n = 248) subjects since the mean YMR is 1.85. As seen in Figure 3C and in Table S9, the survival outcomes of these two groups were significantly different (p = 0.005, HR = 2.63). Similarly, we used YMR cutoff of 1.4 for Bild data set since the mean YMR of the 58 adenocarcinomas is 1.6. The YMR significantly stratified (p = 0.019, HR = 2.72) this independent data set into high (YMR >1.4, n = 31) and low (YMR < = 1.4, n = 27) risk groups (Figure 3B). We calculated the YMR ratio using RNA-seq data of 259 TCGA samples. The continuous YMR scores associate with the survival rate significantly (p-value 0.007, HR 1.87) (Table S9). The dichotomous YMR signature significantly stratified the high- (n = 137) and low-risk (n = 121) groups (p = 0.007, HR = 2.73) (Figure 3D and Table S9).

We calculated the geometric mean of Yin and Yang gene expression ratio (gYMR) and tested its association with the poor outcome both as a continuous variable and a dichotomous variable. As seen in Table S10, the continuous gYMR does not work for Bhattacharjee data and Bild data, and the dichotomous gYMR does not work for Bhattacharjee data either. The arithmetic YMR is robust in four data sets. The continuous YMR did not show its association with clinical outcome in the Bild data set of HG-133plus2 platform (p = 0.49). This is due to the small data size being sensitive to patient outliers or exceptions. After we removed patient GSM70223 whose YMR is 6.35, the p-value of continuous YMR dropped to 0.08. After we further removed patient GSM70159 whose YMR is 2.87 but survived for 73 months, the p-value dropped to a significant level of 0.0199. We do not have sufficient data to help explain why this exception has a high YMR but a long survival time. However these outliers or exceptions did not affect the dichotomous YMR (cutoff >1.4) that significantly stratifies patients’ risk in this data set (p = 0.02, HR = 2.72) (Table S9).

Using the DCC data set, we tested the effect of dropping genes from the Yin and Yang gene list (Figure S6). Dropping one Yin gene (217871_s_at, gene MIF) improved significantly the p-value of YMR, but its HR decreases at the same time (top panel of Figure S6). Dropping one Yin gene affects the p-value of gYMR but did not affect the HR (middle panel of Figure S6). Dropping one Yang gene a time did not affect the p-value of both YMR and gYMR (data not shown), nor the HR of YMR and gYMR (bottom panel of Figure S6). Compared to YMR, gYMR is more resistant to drop-off effect or increased risk association after some genes were dropped. Dropping three Yin genes (HIST1H4J, CDC25A, and IGFBP5) yields best performance of gYMR for DCC data (Middle panel of Figure S6). With the exception of the Bhattacharjee data using dichotomous YMR, the same gene dropping did not improve the performance of either the YMR (Table S11) or the gYMR in other three data sets (Table S12). These results indicate that Yin and Yang gene list could be further optimized to smaller size by removing one to three genes. However, this optimization is constrained by the survival time of the data set tested, similar to the limitations of the data training approach. We expect that around 30 Yin and 30 Yang genes would ensure a representation of the whole Yin and Yang effects of cancer cells and a consistent performance for different data sets. Smaller gene lists may keep the same or improve performance for one data set, but may not work well for other data set.

### Comparison of YMR with Previously Reported Signatures

We compared several aspects of YMR to those of previously reported signatures. As summarized in Table 3, YMR is advanced in reproducibility and practicality. We also compared the prognostic performance of YMR model to a recently reported 15-gene signature [17]. This signature was claimed superior to many other previously reported lung cancer prognostic signatures by testing a same data set with all other signatures. We used the same DCC data set and the Bild adenocarcinoma data [23] from a different platform (U133plus2) for this comparison. As seen in Figure S7A, the 15-gene signature significantly stratified the DCC samples (p = 0.011, HR = 2.68), but not for the Bild samples (Figure S7B, p = 0.6). However, the YMR not only stratified the DCC samples into high risk and low risk groups more significantly (Figure S7C, p = p = 0.005, HR = 2.63) than the 15-gene signature, but also (Figure S7D, p = 0.019, HR = 2.72) separated the Bild samples into the high- and low-risk groups that the 15-gene signature could not. We did not compare the other two data sets (NLCI, Agilent 44k; JBR 10, RT-qPCR) that were used in Zhu *et al* study [17] because these two platforms do not contain enough YMR signature genes. We found the 15-gene signature works best for squamous cell lung carcinomas among all five data sets, but YMR did not work for this data (data not shown), probably due to the difference of tumor biology between squamous cell lung carcinoma and adenocarcinoma.

**Table 3:** Comparison of YMR to different signature models previously reported.

Report	Gene selection reproducibility affected by other cause of survival or false correlation	Signature modeling reproducibility affected by training set intrinsic features	Simplicity of signature delivery	applicable to individual without normalization
Gordon, 2002 [57]	**Yes**. Selection between high and low risk patients	**Yes**, in training two-gene ratio	Simple. Two-gene ratio	**Yes**
Raponi, 2006 [11]	**Yes**. Clusters of genes Cox regression	**Yes**, in building Cox coefficients	Simple equation with trained Cox coefficient	**No**
Chen, 2007 [24]	**Yes**. Cox regression	**Yes**, in building Cox coefficients	Simple equation with trained Cox coefficient	**No**
Shedden, 2008 Method A [13]	**Yes**. Cox regression ofcluster genes	**Yes**, in building penalty likelihood	Complex equation with trained likelihood score	**No**
Zhu, 2010 [17]	**Yes**. Minimum genesfor separation	**Yes**, in building 4 coefficientsfor PCA genes	Moderate complex equation with trained coefficients	**No**
Wan, 2010 [56]	**Yes**. T,SAM,Relief test betweenlong and short survival	**Yes**, in building the priors for Bayesian model	Complex Bayesian equation with trained prior value	**No**
Lu, 2012 [9]	**Yes**. Cox regression	**Yes**, in building Cox coefficients	Moderate complex equation with trained coefficients	**No**
**YMR**	**No**. Expression and function differences between normal and cancer cells	**No**, imbalance of Yin and Yang.No training needed	**Very simple**. Ratio of gene expression	**Yes**

### Analysis of YMR and Clinical Covariates

We evaluated the YMR with clinic covariates in lung cancer prognosis. The 442 DCC samples showed greater than 50% survival rate within 5-year (Figure 3C), which is biased because of the fact that the 5-year overall survival rate for lung cancer is as low as 16% [1]. We used the direct method [53] and only looked at the stage II/III patients within 72 months of follow up time. Not surprisingly, disease stage was the most important risk factor. Excluding disease stage, however, the YMR was the second most important covariate (HR = 1.32, p = 0.006, Table S13). The gYMR signature using actuarial method [53] showed a similar result (HR = 1.67, p = 0.004, Table S14).

**Figure 4:**
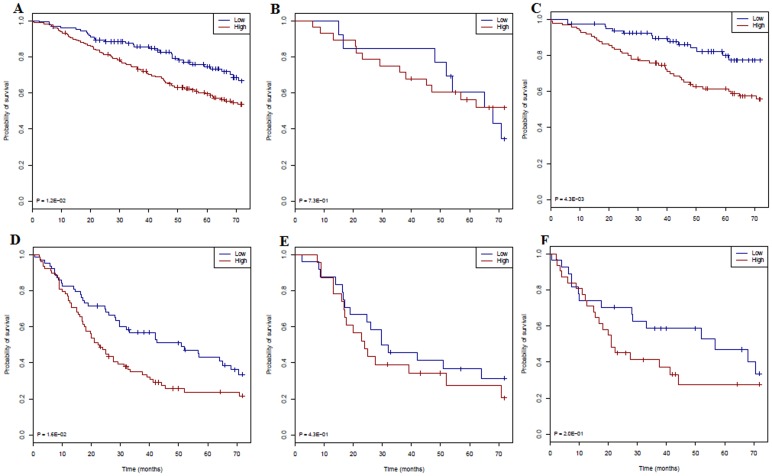
Kaplan-Meier estimates of the survivor function of the gYMR signature in different group of patients of the DCC data set. **A**. Stage I only (low risk n = 122; high risk n = 177). **B**. Stage I who received chemotherapy (low risk n = 13; high risk n = 28). **C**. Stage I who did not receive chemotherapy (low risk n = 79; high risk n = 95). **D**. Stage II & III only (low risk n = 63; high risk n = 78). **E**. Stage II & III who received chemotherapy (low risk n = 24; high risk n = 23). **F**. Stage II & III who did not receive chemotherapy (low risk n = 27; high risk n = 31). Low gYMR scores (in green) correspond to the highest predicted survival probability and high gYMR scores (in red) correspond to the greatest predicted risk.

The YMR stratified 299 stage I DCC patients into high- and low-risk groups (p = 0.048) and 141 stage II/III patients into high- and low-risk groups (p = 0.042). The gYMR risk score showed more significant stratification for stage I patients (Figure 4A, p = 0.012). Unexpectedly, in the whole data set, chemotherapy patients showed even poorer outcome for stage I patients than for those patients who did not receive chemotherapy (Figure S8A). This could be a result from the bias of patient selection for treatment [18]. Conversely, stage II/III patients with high YMR who received chemotherapy showed better outcomes (Figure S8C). For those early stage patients who did not receive chemotherapy, the gYMR risk score was even more significant in predicting prognosis (Figure 4C, p = 0.004). The gYMR score also predicted prognosis among stage II & III patients (p = 0.016) (Figure 4D). These results show that patients with a low-YMR score have a good prognosis regardless of disease stage and chemotherapy can improve outcomes for high-YMR stage II & III patients.

## Discussion

In this study, we developed a new survival prediction signature called YMR for lung cancer. This YMR signature was built from a cancer biology hypothesis in contrast to previously reported models that are based on survival time training (Table 3). The YMR value of individual patients can provide valuable biomarker information relevant to lung cancer prognosis and therapeutic decision-making. In a clinical setting, the ideal prediction model should be applicable to any single patient by providing an informative risk score for that patient. The major shortcoming of all previous prediction models is that the signature gene-expression values of new samples have to be comparable to those of the training sample data in terms of data preprocessing, analysis platform, and data normalization. For example, Shedden K *et al* [13] normalized the entire training and testing data sets together. This is not practical for clinical use. Additionally, global normalization may remove some inter-site differences. Even though using a small number of genes by qRT-PCR [17], [24], [54]–[56] would be more practical, qRT-PCR data also needs to be normalized before the same models can be applied. We propose that YMR not only simplifies the modeling but also avoids data normalization preprocess since the ratio of each patient is comparable. The YMR is computed from the same individuals; therefore, it works for a single patient sample. YMR works for different data analysis platforms and different data preprocess methods. Further, lung cancer prognosis with the YMR could be improved by optimizing the Yin and Yang gene lists and the number of genes in the YMR calculation.

With the advent of microarray technology, groups of differentially expressed genes (DEGs) were chosen between normal tissue samples and cancer samples. To our knowledge, there is no report that selected the DEGs between normal and cancer samples for cancer prognostic signature development. Rather, previous publications selected genes between patients of long and short survival time or genes that correlate to survival time (Table 3). In those publications, Cox regression analysis of all genes against the survival time of all patients resulted in a proportional hazard rate for each gene. The top gene in the list, pre-clustered genes, or metagenes were used as signature genes [9], [11], [13], [17], [24]. Other studies selected genes that were differentially expressed between high-risk and low-risk patients who were simply grouped by survival time [13], [56]. If the same idea (gene association with survival time) was used in gene selection, then the selected signature gene lists would be similar for different studies. Often, however, the published signatures showed little overlap in the genes identified as significant predictors of outcome. Thus, there is a strong possibility that gene selections were influenced by variations in sample collection, sample size, data processing, and microarray platform. Our method does not use survival time as a parameter for gene selection; rather, it used a gene clustering approach instead of group statistics. It is not unexpected that our gene list does not overlap previously reported lung cancer signature genes as our signature development approach is quite different.

The ratio of two-gene expression within an individual patient has been reported as a biomarker signature development in lung cancer diagnosis and prognosis [57]–[58] as well as for breast cancer prognosis [59]–[60]. The single two-gene ratio or geometric mean of several two-gene ratios was selected between the treatment failures and the treatment responders from the training data samples. The single two-gene ratio works well for cancer cell type classification or diagnosis; for example, between malignant pleural mesothelioma (MPM) and adenocarcinoma (ADCA), but it may not be able to reflect the complex tumor progression process for prognosis. In some cases, there could be substantial variation of the two genes among different samples. Therefore many new studies in recent years still rely on the Cox regression modeling to build the prognostic signatures. Most of these models applied the gene expression value to the Cox proportional coefficient of each signature gene and combined them as the patient risk scores [9], [11], [13], [17], [24]. Some models computed the probability of a patient falling into the low-risk or high-risk class as the patient risk scores [56]. However, there are difficulties in using overall survival as an endpoint in prognostic modeling in cancer. The expression variations of the same gene among individual subjects are substantial. Some genes associated with other aggressive diseases may be present in a subject’s tumor. Similarly, a subject might develop and succumb to some other clinical condition shortly after diagnosis. In these instances, no correlation exists between gene expression and subject survival [18]. The complex models could learn the expression variable as well as other variations precisely, which would result in low reproducibility if used for a different data set. Instead, we return to the two-group gene expression ratio approach but select these two groups of genes using differences between normal lung cells and lung cancer cells that represent the whole Yin and Yang effects of the cell. Among the Yin genes we selected included pathways and networks connected to the canonical Molecular Mechanisms of Cancer pathway. The Yang genes are connected to the Retinoic acid receptor (RAR) activation pathway and the Hepatic Stellate Cell Activation pathway. RAR activation induces cell differentiation and may antagonize cancer progression because retinoic acid or vitamin A is required for the differentiated state of normal cells [46]. Hepatic Stellate Cells play a key role in the storage and transport of retinoids and the lung tissue harbors the Hepatic Stellate-like cells [46]–[47]. These two pathways alter the balance between Yin and Yang, consequently altering patient survival.

A useful prognostic signature should not only predict the patient's prognosis, but should also help clinical therapeutic decision making. Although surgery is a standard treatment for early stage lung cancer, more than 20% of stage I patients will relapse. This cohort of patients may benefit from chemotherapy. For the late stage lung cancer patients, after the complete resection of tumors, a good prognostic signal could spare the patient from chemotherapy or recommend less intensive therapy. Our YMR signature shows potential in helping clinical therapeutic decision making for different stages of lung cancer. For those high-YMR stage I patients, a careful therapy recipe is recommended. Chemotherapy can improve outcomes for high-YMR stage II & III patients.

### Conclusions

In this study, we developed a novel biomarker signature (YMR) for lung cancer. YMR signature was based on a Yin Yang hypothesis that the imbalance of two opposing effects in lung cells determines the patient's prognosis. This contrasts with all previous signature models that were based on significant data training. This study provided a novel insight into the biology of lung cancer development. Experimental validation of this approach and a qRT-PCR kit designed for the Yin and Yang gene expression level detection are the next steps. The calculated YMR risk score can help the clinical therapy decision making regarding the disease stages. This study can also have potential in drug development by modulating the Yin and Yang (Figure 1B) or altering other target gene expression so that a lower YMR can be achieved. In addition to lung cancer, the YMR approach to biomarker discovery could be applied to other cancers or diseases as well.

## Supporting Information

Figure S1 **2-D clustering for identification of Yin gene candidates. A**. 2-D Euclidean clustering with complete linkage setting for both gene (12,625 genes on HG-U95av2) and 100 samples of Bhattacharjee data set. The region was selected where the genes downregulated in normal samples but upregulated in almost all different types of lung cancers. The region where genes were upregulated in one or few cancer types was not selected. **B**. The selected region was zoomed in from the whole array view.
(TIF)

Figure S2 **2-D clustering for identification of Yang gene candidates. A**. 2-D Euclidean clustering with complete linkage setting for both gene (12,625 genes on HG-U95av2) and 100 samples of Bhattacharjee data set. The region was selected where the genes upregulated in normal samples but downregulated in almost all different types of lung cancers. The region where genes were downregulated in one or few cancer types was not selected. **B**. The selected region was zoomed in from the whole array view.
(TIF)

Figure S3 **74 Yin gene probe sets were analyzed by using IPA.** The Molecular Mechanisms of cancer canonical pathway was highlighted by green lines.
(TIF)

Figure S4 **Top protein interaction network and pathway of Yang genes.** 108 Yang gene probe sets were analyzed by using IPA. The RAR Activation pathway and the Hepatic Stellate Cell Activation pathway were highlighted by green lines.
(TIF)

Figure S5 **Random group gene expression ratios.** 500 groups of 31 genes and 500 groups of 32 genes randomly picked up from 12,625 genes among 125 Adenocarcinomas **of** Bhattacharjee data set. **A**. Histogram of 500 p-values of random group ratios as continuous variable. **B**. Histogram of 500 hazard Ratios (HR) of random group ratios as continuous variable. **C.** Histogram of 500 p-values of random group ratios as dichotomous (ratio >2.0) variable. **D**. Histogram of 500 hazard Ratios (HR) of random group ratios as dichotomous (ratio >2.0) variable. The stratification of these 500 random ratios (>2.0) was tested by Cox proportional hazard ratio model.
(TIF)

Figure S6 **Effect of dropping genes from YMR signature gene list.** The continuous YMR and gYMR were tested on 442 samples of DCC data set after one or more genes were removed from the 63 Yin and Yang gene list. **Upper panel**: The effect on YMR of dropping Yin genes. **A**. “orig” is the original 31 yin gene, dropping one gene a time, dropping two genes (“24_10″, i.e. HIST1H4J, 214463_x; CDC25A, 204696_s), as well as dropping three genes (24-10-7, i.e. HIST1H4J; CDC25A; and IGFBP5, 203425_s). These three genes were chosen because they showed best performance in gYMR after they were dropped. **B**. The effect on HR using the same genes as in A. **Middle panel**: The effect on gYMR of dropping Yin genes. “orig” is the original 31 yin gene, dropping one gene a time, dropping two genes (24-10, i.e. HIST1H4J, CDC25A), as well as dropping three genes (24-10-7, i.e. HIST1H4J, CDC25A, and IGFBP5). **Lower panel**: The effect on HR of dropping Yang genes. **A**. The effect on YMR. “orig” is the original 32 yang gene, and dropping one gene a time. **B**. The effect on gYMR. “orig”, the original 32 yang gene, or dropping one gene a time.
(TIF)

Figure S7 **Comparing YMR to the 15-gene signature. A**. 15-gene signature (Zhu *et al* 2010) for the DCC sample data (low = 231; high = 211). **B**. 15-gene signature for Bild data (low = 35; high = 23). **C**. YMR for the same DCC sample data (low = 248; high = 195). **D**. YMR for the same Bild data (low = 27; high = 31).
(TIF)

Figure S8 **Kaplan-Meier estimates of the survivor function of patients with or without chemotherapy after diagnosis.** **A**. All stage I patient samples from the DCC project. **B**. Low YMR stage II&III patients. **C**. High YMR stage II&III patients.
(TIF)

Table S1 **74 Yin genes.**
(DOC)

Table S2 **108 Yang genes.**
(DOC)

Table S3 **Yin gene network 1.** Network that is related to tumor morphology (network significant score of 42) was selected from pathway and interaction network analyses of these 74.
(XLS)

Table S4 **Yin gene network 2.** Network that is related to DNA replication (network significant score of 30) was selected from pathway and interaction network analyses of these 74 Yin genes.
(XLS)

Table S5 **Yang genes involved cellular development.** The focus genes related to cellular maintenance process were extracted from IPA.
(XLS)

Table S6 **Yang genes involved maintenance process.** The focus genes related to cellular development process were extracted from IPA.
(XLS)

Table S7 **Data sets and preprocesses used in this study.**
(DOC)

Table S8 **YMR values of the different normal tissue types.**
(DOC)

Table S9 **Continuous and dichotomous arithmetic YMR scores are associated with clinical outcomes.**
(DOC)

Table S10 **Continuous and dichotomous geometric gYMR scores are associated with clinical outcomes.**
(DOC)

Table S11 **Dropping three genes (HIST1H4J, CDC25A, and IGFBP5) on continuous and dichotomous YMR.**
(DOC)

Table S12 **Dropping three genes (HIST1H4J, CDC25A, and IGFBP5) on continuous and dichotomous gYMR.**
(DOC)

Table S13 **YMR covariate and multivariate analysis using direct method** [**53**] **.**
(DOC)

Table S14 **gYMR covariate and multivariate analysis using actuarial method** [**53**] **.**
(DOC)
