# Supplementary figures and images for: Yin Yang Gene Expression Ratio Signature for Lung Cancer Prognosis
Source: PLoS One. 2013 Jul 17;8(7):e68742. doi: 10.1371/journal.pone.0068742 (PMC3714286; doi:10.1371/journal.pone.0068742)

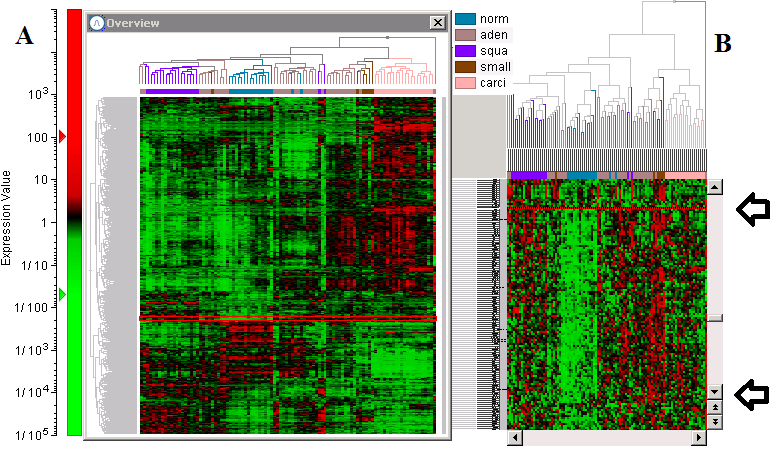

Supplement: Figure S1 — 2-D clustering for identification of Yin gene candidates. A. 2-D Euclidean clustering with complete linkage setting for both gene (12,625 genes on HG-U95av2) and 100 samples of Bhattacharjee data set. The region was selected where the genes downregulated in normal samples but upregulated in almost all different types of lung cancers. The region where genes were upregulated in one or few cancer types was not selected. B. The selected region was zoomed in from the whole array view. (TIF) [file pone.0068742.s001.tif]

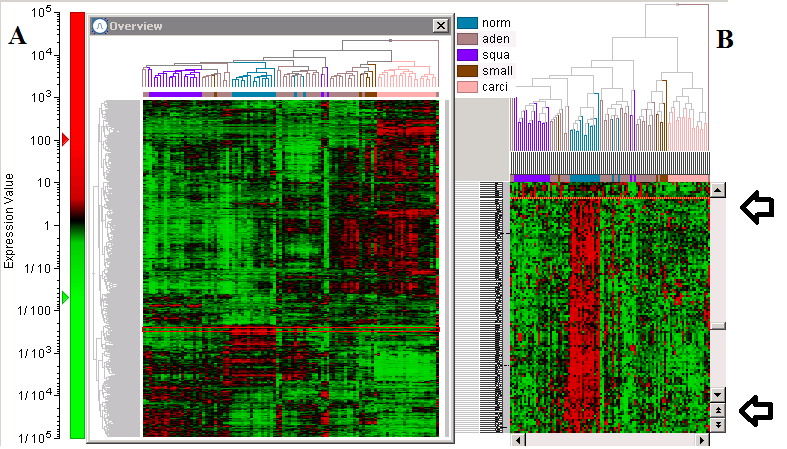

Supplement: Figure S2 — 2-D clustering for identification of Yang gene candidates. A. 2-D Euclidean clustering with complete linkage setting for both gene (12,625 genes on HG-U95av2) and 100 samples of Bhattacharjee data set. The region was selected where the genes upregulated in normal samples but downregulated in almost all different types of lung cancers. The region where genes were downregulated in one or few cancer types was not selected. B. The selected region was zoomed in from the whole array view. (TIF) [file pone.0068742.s002.tif]

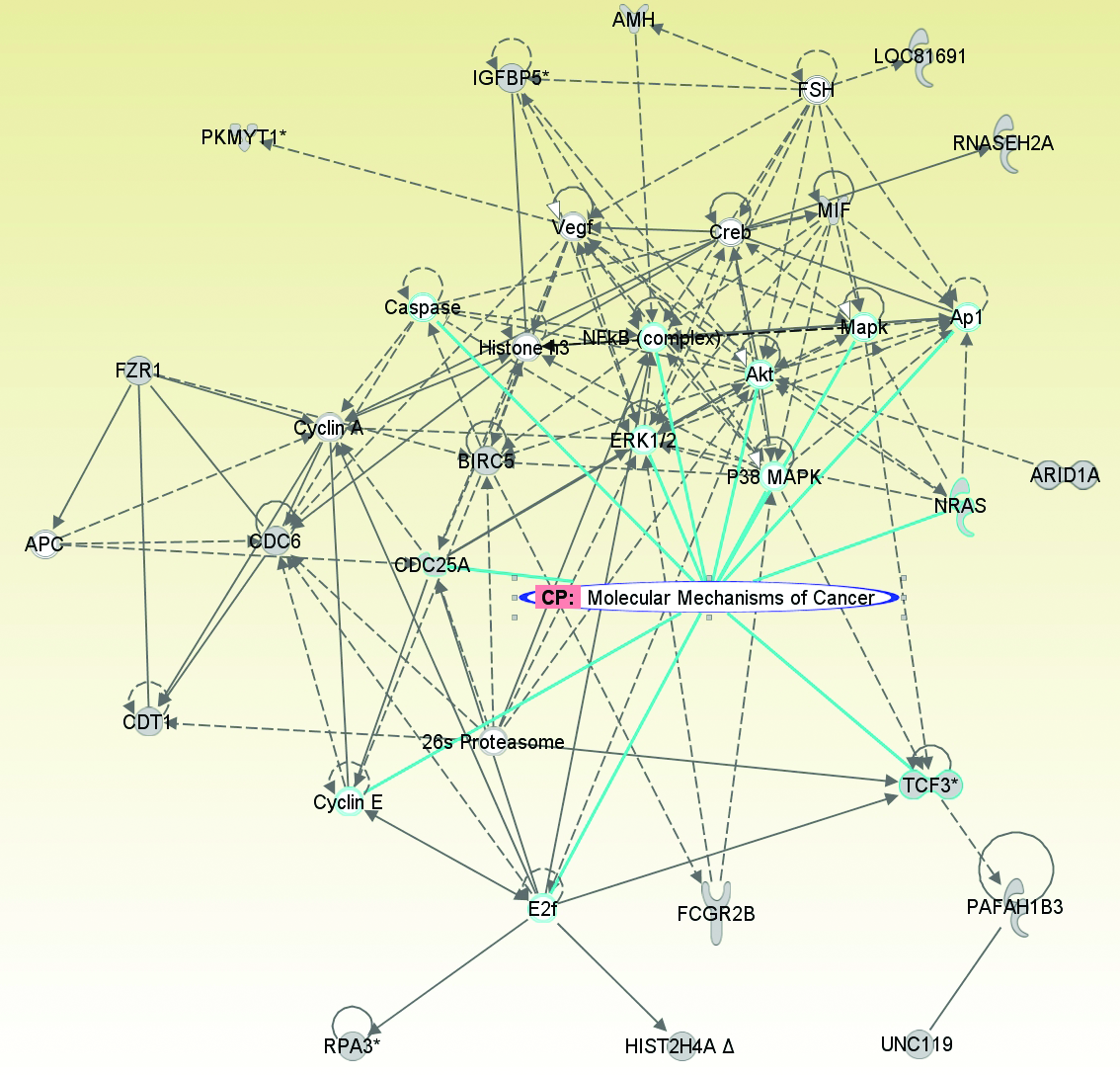

Supplement: Figure S3 — 74 Yin gene probe sets were analyzed by using IPA. The Molecular Mechanisms of cancer canonical pathway was highlighted by green lines. (TIF) [file pone.0068742.s003.tif]

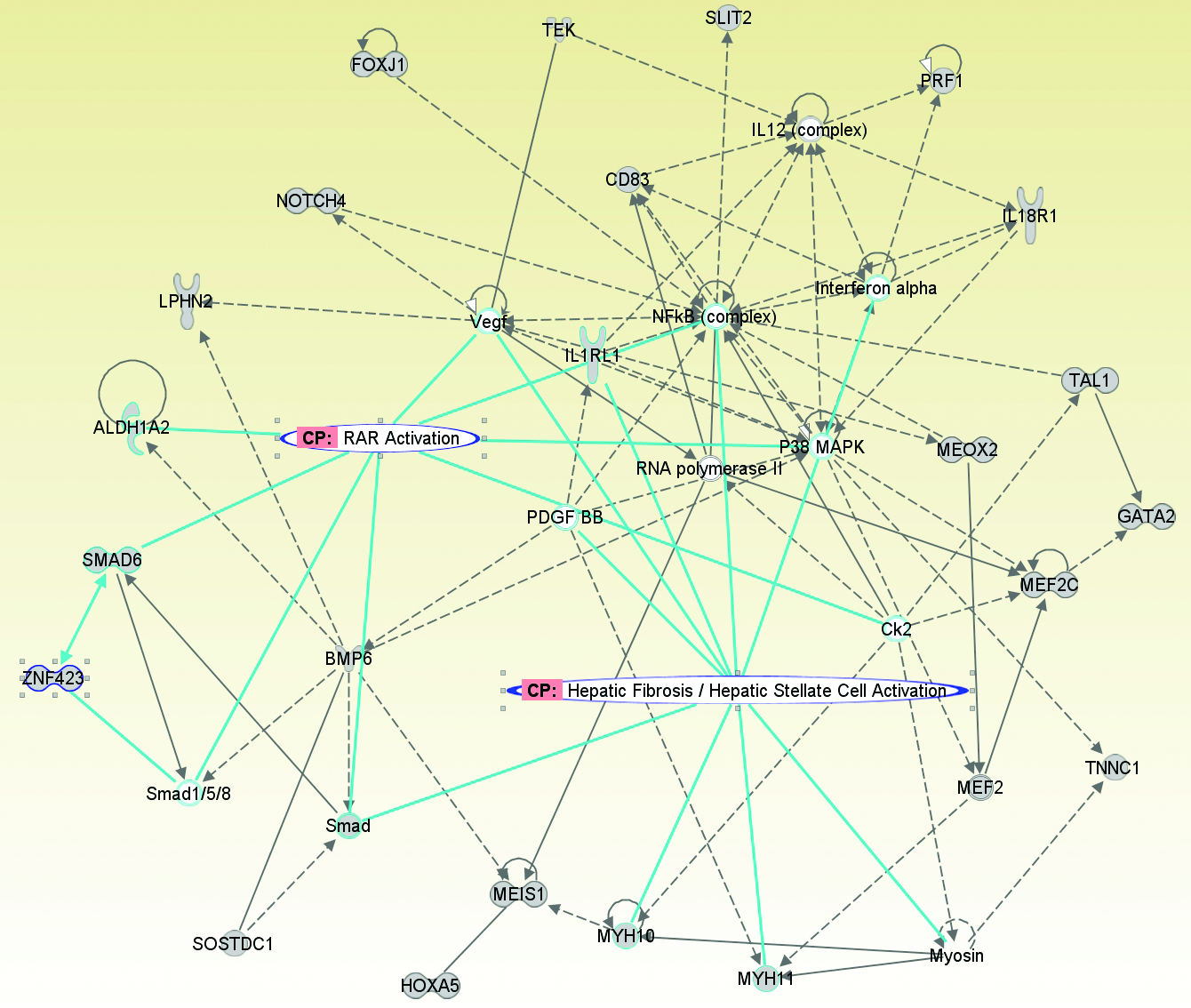

Supplement: Figure S4 — Top protein interaction network and pathway of Yang genes. 108 Yang gene probe sets were analyzed by using IPA. The RAR Activation pathway and the Hepatic Stellate Cell Activation pathway were highlighted by green lines. (TIF) [file pone.0068742.s004.tif]

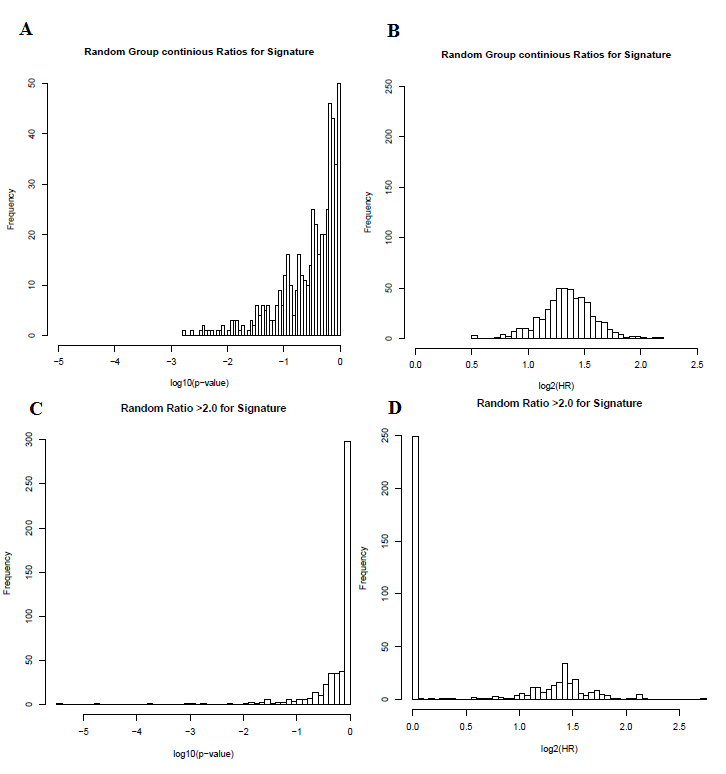

Supplement: Figure S5 — Random group gene expression ratios. 500 groups of 31 genes and 500 groups of 32 genes randomly picked up from 12,625 genes among 125 Adenocarcinomas of Bhattacharjee data set. A. Histogram of 500 p-values of random group ratios as continuous variable. B. Histogram of 500 hazard Ratios (HR) of random group ratios as continuous variable. C. Histogram of 500 p-values of random group ratios as dichotomous (ratio >2.0) variable. D. Histogram of 500 hazard Ratios (HR) of random group ratios as dichotomous (ratio >2.0) variable. The stratification of these 500 random ratios (>2.0) was tested by Cox proportional hazard ratio model. (TIF) [file pone.0068742.s005.tif]

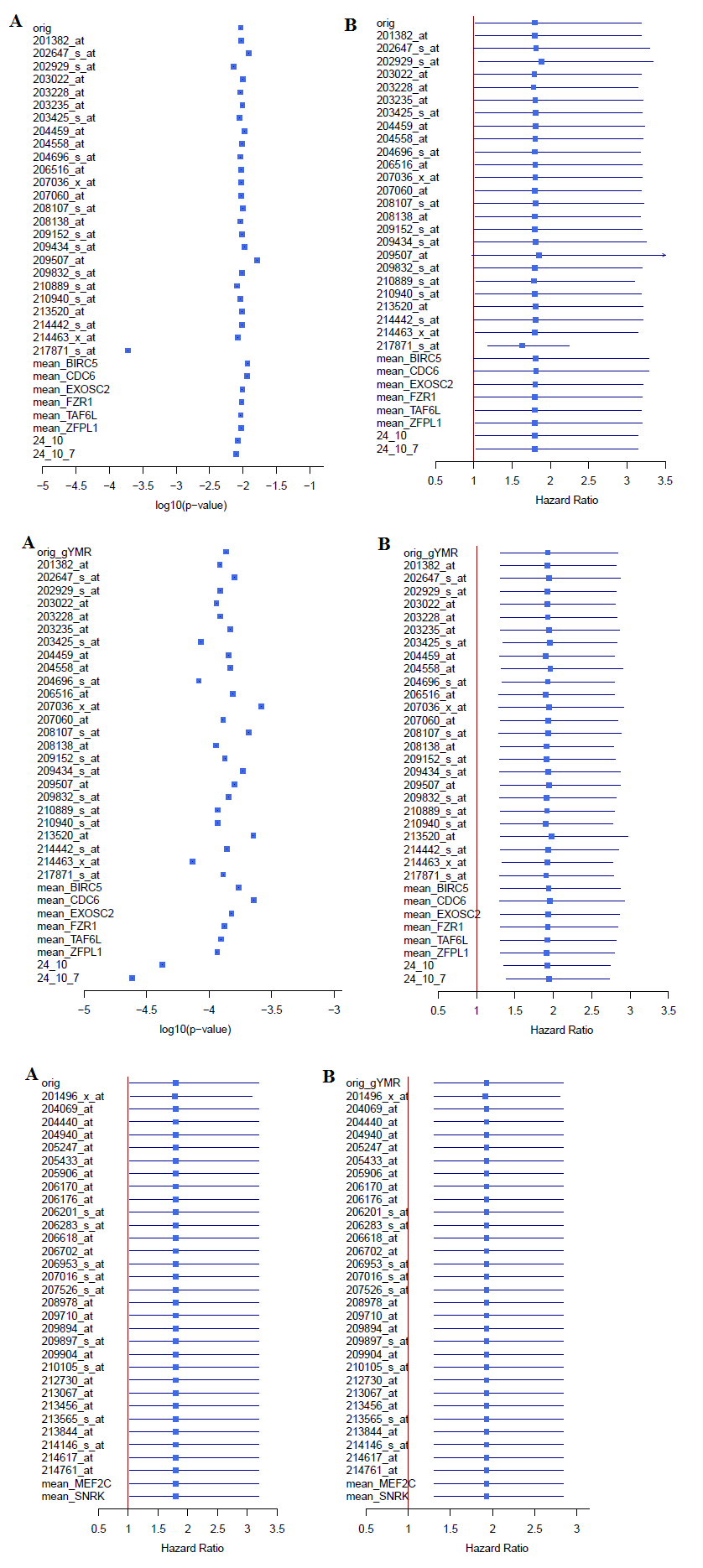

Supplement: Figure S6 — Effect of dropping genes from YMR signature gene list. The continuous YMR and gYMR were tested on 442 samples of DCC data set after one or more genes were removed from the 63 Yin and Yang gene list. Upper panel: The effect on YMR of dropping Yin genes. A. “orig” is the original 31 yin gene, dropping one gene a time, dropping two genes (“24_10″, i.e. HIST1H4J, 214463_x; CDC25A, 204696_s), as well as dropping three genes (24-10-7, i.e. HIST1H4J; CDC25A; and IGFBP5, 203425_s). These three genes were chosen because they showed best performance in gYMR after they were dropped. B. The effect on HR using the same genes as in A. Middle panel: The effect on gYMR of dropping Yin genes. “orig” is the original 31 yin gene, dropping one gene a time, dropping two genes (24-10, i.e. HIST1H4J, CDC25A), as well as dropping three genes (24-10-7, i.e. HIST1H4J, CDC25A, and IGFBP5). Lower panel: The effect on HR of dropping Yang genes. A. The effect on YMR. “orig” is the original 32 yang gene, and dropping one gene a time. B. The effect on gYMR. “orig”, the original 32 yang gene, or dropping one gene a time. (TIF) [file pone.0068742.s006.tif]

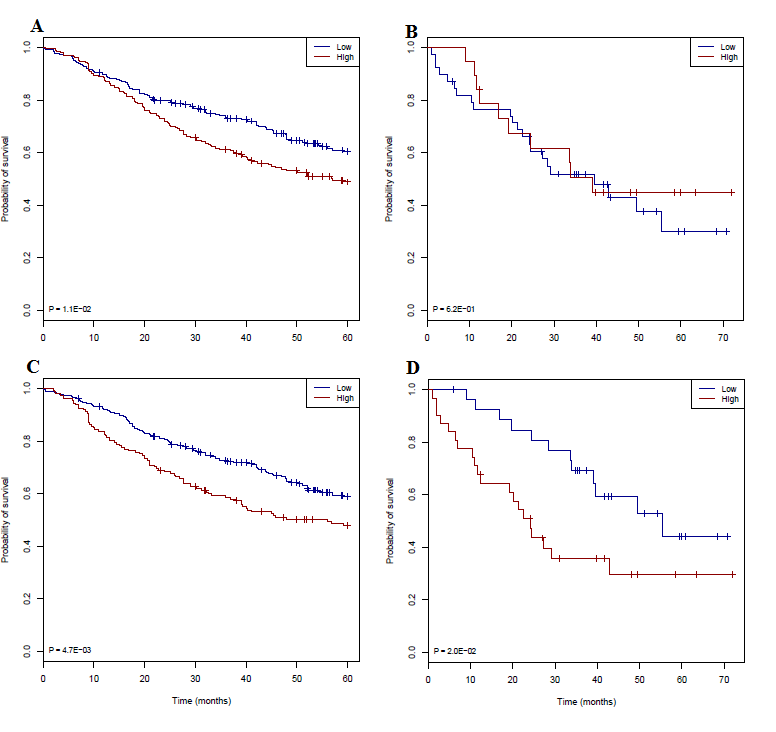

Supplement: Figure S7 — Comparing YMR to the 15-gene signature. A. 15-gene signature (Zhu et al 2010) for the DCC sample data (low = 231; high = 211). B. 15-gene signature for Bild data (low = 35; high = 23). C. YMR for the same DCC sample data (low = 248; high = 195). D. YMR for the same Bild data (low = 27; high = 31). (TIF) [file pone.0068742.s007.tif]

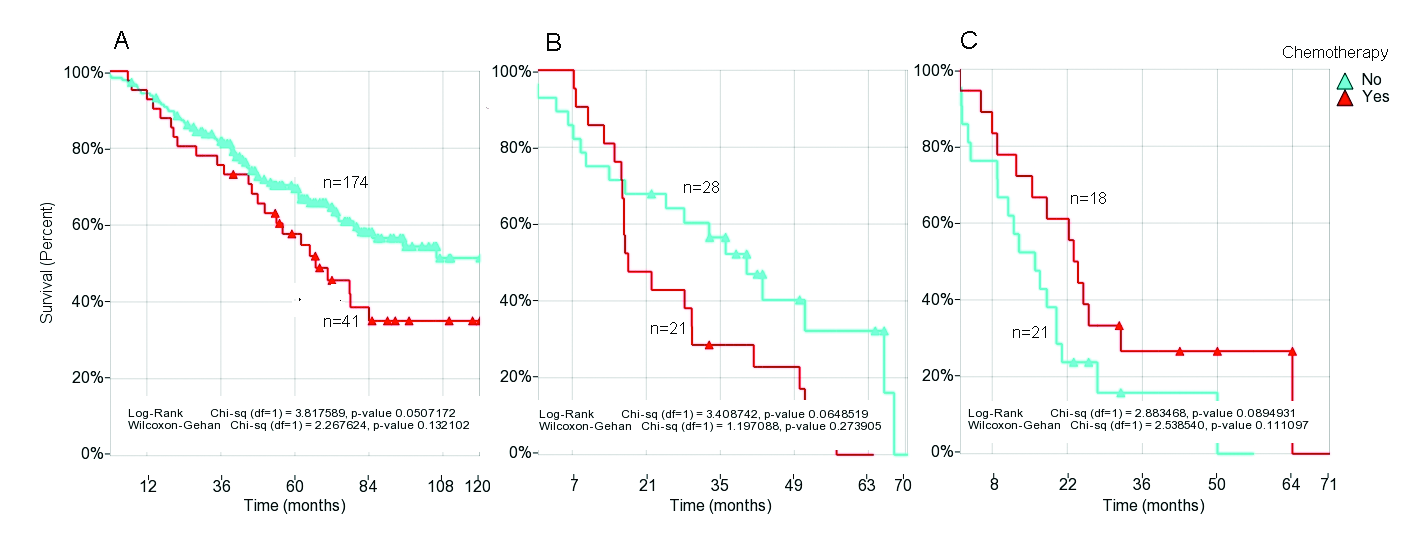

Supplement: Figure S8 — Kaplan-Meier estimates of the survivor function of patients with or without chemotherapy after diagnosis. A. All stage I patient samples from the DCC project. B. Low YMR stage II&III patients. C. High YMR stage II&III patients. (TIF) [file pone.0068742.s008.tif]
