# Supplementary material for: Yin Yang Gene Expression Ratio Signature for Lung Cancer Prognosis
Source: PLoS One. 2013 Jul 17;8(7):e68742. doi: 10.1371/journal.pone.0068742 (PMC3714286; doi:10.1371/journal.pone.0068742)
Supplement: Table S1 — 74 Yin genes. (DOC) [file pone.0068742.s009.doc]

**Table S1**. 74 Yin genes

| Probe set | Gene Title | Gene Symbol | Entrez Gene |
| --- | --- | --- | --- |
| 31353_f_at | forkhead box E1 (thyroid transcription factor 2) | FOXE1 | 2304 |
| 31521_f_at | histone cluster 1, H4j /// histone cluster 1, H4k | HIST1H4J /// HIST1H4K | 8362 /// 8363 |
| 31591_s_at | complement factor H-related 4 | CFHR4 | 10877 |
| 31631_f_at | pituitary tumor-transforming 2 | PTTG2 | 10744 |
| 31711_at | glutamate receptor, ionotropic, N-methyl D-aspartate 2D | GRIN2D | 2906 |
| 31997_at | --- | --- | --- |
| 32449_at | diacylglycerol kinase, theta 110kDa | DGKQ | 1609 |
| 34096_at | centrosomal protein 152kDa | CEP152 | 22995 |
| 34552_at | gastrin | GAST | 2520 |
| 35084_at | anti-Mullerian hormone | AMH | 268 |
| 35504_at | AT rich interactive domain 1A (SWI-like) | ARID1A | 8289 |
| 36402_at | zinc finger protein 101 | ZNF101 | 94039 |
| 36418_at | prickle homolog 3 (Drosophila) | PRICKLE3 | 4007 |
| 32874_at | transcription factor 3 (E2A immunoglobulin enhancer binding factors E12/E47) | TCF3 | 6929 |
| 32975_g_at | exosome component 2 | EXOSC2 | 23404 |
| 33510_s_at | glutamate receptor, metabotropic 1 | GRM1 | 2911 |
| 34027_f_at | histone cluster 1, H4j /// histone cluster 1, H4k | HIST1H4J /// HIST1H4K | 8362 /// 8363 |
| 34510_at | chromatin licensing and DNA replication factor 1 | CDT1 | 81620 |
| 35375_at | APEX nuclease (apurinic/apyrimidinic endonuclease) 2 | APEX2 | 27301 |
| 35401_s_at | matrix metallopeptidase 17 (membrane-inserted) | MMP17 | 4326 |
| 36691_at | cysteine conjugate-beta lyase, cytoplasmic | CCBL1 | 883 |
| 37094_at | X-ray repair complementing defective repair in Chinese hamster cells 3 | XRCC3 | 7517 |
| 37424_at | coiled-coil alpha-helical rod protein 1 | CCHCR1 | 54535 |
| 37432_g_at | protein inhibitor of activated STAT, 2 | PIAS2 | 9063 |
| 37872_at | jerky homolog (mouse) | JRK | 8629 |
| 37873_g_at | jerky homolog (mouse) | JRK | 8629 |
| 38184_at | paired box 2 | PAX2 | 5076 |
| 39262_at | SAC3 domain containing 1 | SAC3D1 | 29901 |
| 39651_at | RecQ protein-like 4 | RECQL4 | 9401 |
| 40334_at | cleavage stimulation factor, 3' pre-RNA, subunit 2, 64kDa | CSTF2 | 1478 |
| 41374_at | ribosomal protein S6 kinase, 70kDa, polypeptide 2 | RPS6KB2 | 6199 |
| 41420_at | insulin-like growth factor binding protein 5 | IGFBP5 | 3488 |
| 41623_s_at | fizzy/cell division cycle 20 related 1 (Drosophila) | FZR1 | 51343 |
| 41664_at | translocase of inner mitochondrial membrane 44 homolog (yeast) | TIMM44 | 10469 |
| 34664_at | Fc fragment of IgG, low affinity IIb, receptor (CD32) | FCGR2B | 2213 |
| 35141_at | ribonuclease H2, subunit A | RNASEH2A | 10535 |
| 36839_at | cell division cycle 6 homolog (S. cerevisiae) | CDC6 | 990 |
| 37238_s_at | protein kinase, membrane associated tyrosine/threonine 1 | PKMYT1 | 9088 |
| 37267_at | thimet oligopeptidase 1 | THOP1 | 7064 |
| 40794_at | kallikrein-related peptidase 3 | KLK3 | 354 |
| 40866_at | nipsnap homolog 1 (C. elegans) | NIPSNAP1 | 8508 |
| 41149_at | exonuclease NEF-sp | LOC81691 | 81691 |
| 32213_at | processing of precursor 7, ribonuclease P/MRP subunit (S. cerevisiae) | POP7 | 10248 |
| 33935_at | calcyclin binding protein | CACYBP | 27101 |
| 34341_at | phosphoribosyl pyrophosphate amidotransferase | PPAT | 5471 |
| 35800_at | platelet-activating factor acetylhydrolase 1b, catalytic subunit 3 (29kDa) | PAFAH1B3 | 5050 |
| 38107_at | unc-119 homolog (C. elegans) | UNC119 | 9094 |
| 38373_g_at | chromosome X open reading frame 40A /// chromosome X open reading frame 40B | CXorf40A /// CXorf40B | 541578 /// 91966 |
| 38401_s_at | LSM14A, SCD6 homolog A (S. cerevisiae) | LSM14A | 26065 |
| 39909_g_at | TAF6-like RNA polymerase II, p300/CBP-associated factor (PCAF)-associated factor, 65kDa | TAF6L | 10629 |
| 40263_at | zinc finger protein-like 1 | ZFPL1 | 7542 |
| 40264_g_at | zinc finger protein-like 1 | ZFPL1 | 7542 |
| 40528_at | LIM homeobox 2 | LHX2 | 9355 |
| 40532_at | baculoviral IAP repeat-containing 5 | BIRC5 | 332 |
| 40597_g_at | Treacher Collins-Franceschetti syndrome 1 | TCOF1 | 6949 |
| 40891_f_at | L antigen family, member 3 | LAGE3 | 8270 |
| 41851_at | Coiled-coil domain containing 85B | CCDC85B | 11007 |
| 32559_s_at | LSM4 homolog, U6 small nuclear RNA associated (S. cerevisiae) | LSM4 | 25804 |
| 33203_s_at | forkhead box D1 | FOXD1 | 2297 |
| 1738_at | cell division cycle 25 homolog A (S. pombe) | CDC25A | 993 |
| 1678_g_at | insulin-like growth factor binding protein 5 | IGFBP5 | 3488 |
| 1601_s_at | insulin-like growth factor binding protein 5 | IGFBP5 | 3488 |
| 1539_at | neuroblastoma RAS viral (v-ras) oncogene homolog | NRAS | 4893 |
| 1374_g_at | transcription factor 3 (E2A immunoglobulin enhancer binding factors E12/E47) | TCF3 | 6929 |
| 1133_at | engrailed homeobox 2 | EN2 | 2020 |
| 966_at | RAD54-like (S. cerevisiae) | RAD54L | 8438 |
| 895_at | macrophage migration inhibitory factor (glycosylation-inhibiting factor) | MIF | 4282 |
| 762_f_at | histone cluster 1, H4i | HIST1H4I | 8294 |
| 696_at | homeobox D8 | HOXD8 | 3234 |
| 651_at | replication protein A3, 14kDa | RPA3 | 6119 |
| 652_g_at | replication protein A3, 14kDa | RPA3 | 6119 |
| 480_at | protein kinase, membrane associated tyrosine/threonine 1 | PKMYT1 | 9088 |
| 374_f_at | D-dopachrome tautomerase /// D-dopachrome tautomerase-like | DDT /// DDTL | 100037417 /// 1652 |
| 152_f_at | histone cluster 2, H4a /// histone cluster 2, H4b | HIST2H4A /// HIST2H4B | 554313 /// 8370 |
