# Supplementary material for: Yin Yang Gene Expression Ratio Signature for Lung Cancer Prognosis
Source: PLoS One. 2013 Jul 17;8(7):e68742. doi: 10.1371/journal.pone.0068742 (PMC3714286; doi:10.1371/journal.pone.0068742)
Supplement: Table S2 — 108 Yang genes. (DOC) [file pone.0068742.s010.doc]

**Table S2**. 108 Yang genes

| Probe set | Gene Title | Gene Symbol | Entrez Gene |
| --- | --- | --- | --- |
| 31506_s_at | defensin, alpha 1 /// defensin, alpha 1B /// defensin, alpha 3, neutrophil-specific | DEFA1 /// DEFA1B /// DEFA3 | 1667 /// 1668 /// 728358 |
| 33690_at | --- | --- | --- |
| 34174_s_at | latrophilin 2 | LPHN2 | 23266 |
| 34604_at | solute carrier family 6 (neurotransmitter transporter, serotonin), member 4 | SLC6A4 | 6532 |
| 35079_at | contactin 6 | CNTN6 | 27255 |
| 35606_at | histidine decarboxylase | HDC | 3067 |
| 36377_at | interleukin 18 receptor 1 | IL18R1 | 8809 |
| 32370_at | granzyme H (cathepsin G-like 2, protein h-CCPX) | GZMH | 2999 |
| 32904_at | perforin 1 (pore forming protein) | PRF1 | 5551 |
| 32971_at | family with sequence similarity 189, member A2 | FAM189A2 | 9413 |
| 33462_at | purinergic receptor P2Y, G-protein coupled, 14 | P2RY14 | 9934 |
| 34940_at | --- | --- | --- |
| 34950_at | zinc finger protein 423 | ZNF423 | 23090 |
| 34965_at | cystatin F (leukocystatin) | CST7 | 8530 |
| 35964_at | matrilin 3 | MATN3 | 4148 |
| 36258_at | protein kinase, cGMP-dependent, type I | PRKG1 | 5592 |
| 36275_at | sema domain, transmembrane domain (TM), and cytoplasmic domain, (semaphorin) 6A | SEMA6A | 57556 |
| 37121_at | natural killer cell group 7 sequence | NKG7 | 4818 |
| 37154_at | protocadherin 17 | PCDH17 | 27253 |
| 37841_at | butyrylcholinesterase | BCHE | 590 |
| 39208_i_at | pro-platelet basic protein (chemokine (C-X-C motif) ligand 7) | PPBP | 5473 |
| 39209_r_at | pro-platelet basic protein (chemokine (C-X-C motif) ligand 7) | PPBP | 5473 |
| 39279_at | bone morphogenetic protein 6 | BMP6 | 654 |
| 39325_at | left-right determination factor 2 | LEFTY2 | 7044 |
| 39577_at | sclerostin domain containing 1 | SOSTDC1 | 25928 |
| 39634_at | slit homolog 2 (Drosophila) | SLIT2 | 9353 |
| 39673_i_at | extracellular matrix protein 2, female organ and adipocyte specific | ECM2 | 1842 |
| 39674_r_at | extracellular matrix protein 2, female organ and adipocyte specific | ECM2 | 1842 |
| 40034_r_at | scavenger receptor class F, member 1 | SCARF1 | 8578 |
| 40322_at | interleukin 1 receptor-like 1 | IL1RL1 | 9173 |
| 40374_at | ankyrin repeat domain 1 (cardiac muscle) | ANKRD1 | 27063 |
| 40398_s_at | mesenchyme homeobox 2 | MEOX2 | 4223 |
| 40399_r_at | mesenchyme homeobox 2 | MEOX2 | 4223 |
| 40665_at | flavin containing monooxygenase 3 | FMO3 | 2328 |
| 40739_at | carbonic anhydrase IV | CA4 | 762 |
| 41013_at | chromosome 10 open reading frame 72 | C10orf72 | 196740 |
| 41030_at | forkhead box J1 | FOXJ1 | 2302 |
| 41644_at | SAM and SH3 domain containing 1 | SASH1 | 23328 |
| 31892_at | protein tyrosine phosphatase, receptor type, M | PTPRM | 5797 |
| 32740_at | RAB11 family interacting protein 2 (class I) | RAB11FIP2 | 22841 |
| 33328_at | HEG homolog 1 (zebrafish) | HEG1 | 57493 |
| 33766_at | vasoactive intestinal peptide receptor 1 | VIPR1 | 7433 |
| 34203_at | calponin 1, basic, smooth muscle | CNN1 | 1264 |
| 34267_r_at | leptin receptor | LEPR | 3953 |
| 35234_at | reversion-inducing-cysteine-rich protein with kazal motifs | RECK | 8434 |
| 35985_at | A kinase (PRKA) anchor protein 2 /// PALM2-AKAP2 readthrough | AKAP2 /// PALM2-AKAP2 | 11217 /// 445815 |
| 36061_at | sema domain, seven thrombospondin repeats (type 1 and type 1-like), transmembrane domain (TM) and short cytoplasmic domain, (semaphorin) 5A | SEMA5A | 9037 |
| 36915_at | cathepsin O | CTSO | 1519 |
| 37194_at | GATA binding protein 2 | GATA2 | 2624 |
| 37251_s_at | glycoprotein M6B | GPM6B | 2824 |
| 37253_at | phosphatidylinositol-4-phosphate 5-kinase, type I, beta | PIP5K1B | 8395 |
| 37536_at | CD83 molecule | CD83 | 9308 |
| 37958_at | transmembrane protein 47 | TMEM47 | 83604 |
| 38315_at | aldehyde dehydrogenase 1 family, member A2 | ALDH1A2 | 8854 |
| 39031_at | cytochrome c oxidase subunit VIIa polypeptide 1 (muscle) | COX7A1 | 1346 |
| 39048_at | Notch homolog 4 (Drosophila) | NOTCH4 | 4855 |
| 39085_at | troponin C type 1 (slow) | TNNC1 | 7134 |
| 39356_at | neural precursor cell expressed, developmentally down-regulated 4-like | NEDD4L | 23327 |
| 39400_at | TBC1 domain family, member 2B | TBC1D2B | 23102 |
| 39750_at | --- | --- | --- |
| 40434_at | podocalyxin-like | PODXL | 5420 |
| 40480_s_at | FYN oncogene related to SRC, FGR, YES | FYN | 2534 |
| 40763_at | Meis homeobox 1 | MEIS1 | 4211 |
| 41151_at | inositol polyphosphate-5-phosphatase K | INPP5K | 51763 |
| 32208_at | KIAA0355 | KIAA0355 | 9710 |
| 32838_at | myosin, heavy chain 10, non-muscle | MYH10 | 4628 |
| 33872_at | latrophilin 2 | LPHN2 | 23266 |
| 35344_at | LIM domain 7 | LMO7 | 4008 |
| 35828_at | cysteine-rich protein 2 | CRIP2 | 1397 |
| 36577_at | fermitin family homolog 2 (Drosophila) | FERMT2 | 10979 |
| 36627_at | SPARC-like 1 (hevin) | SPARCL1 | 8404 |
| 36939_at | glycoprotein M6A | GPM6A | 2823 |
| 37407_s_at | myosin, heavy chain 11, smooth muscle | MYH11 | 4629 |
| 37710_at | myocyte enhancer factor 2C | MEF2C | 4208 |
| 37718_at | SNF related kinase | SNRK | 54861 |
| 38734_at | phospholamban | PLN | 5350 |
| 38747_at | CD34 molecule | CD34 | 947 |
| 38748_at | adenosine deaminase, RNA-specific, B1 (RED1 homolog rat) | ADARB1 | 104 |
| 39452_s_at | spectrin, beta, non-erythrocytic 1 | SPTBN1 | 6711 |
| 39541_at | HEG homolog 1 (zebrafish) | HEG1 | 57493 |
| 39544_at | synemin, intermediate filament protein | SYNM | 23336 |
| 40231_at | SMAD family member 6 | SMAD6 | 4091 |
| 40900_at | myosin, heavy chain 10, non-muscle | MYH10 | 4628 |
| 40971_at | ankyrin repeat and sterile alpha motif domain containing 1A | ANKS1A | 23294 |
| 40994_at | G protein-coupled receptor kinase 5 | GRK5 | 2869 |
| 41549_s_at | adaptor-related protein complex 1, sigma 2 subunit | AP1S2 | 8905 |
| 41837_at | chromosome 14 open reading frame 132 | C14orf132 | 56967 |
| 32582_at | myosin, heavy chain 11, smooth muscle | MYH11 | 4629 |
| 32593_at | raftlin, lipid raft linker 1 | RFTN1 | 23180 |
| 2039_s_at | FYN oncogene related to SRC, FGR, YES | FYN | 2534 |
| 1733_at | bone morphogenetic protein 6 | BMP6 | 654 |
| 1595_at | TEK tyrosine kinase, endothelial | TEK | 7010 |
| 1389_at | membrane metallo-endopeptidase | MME | 4311 |
| 1135_at | G protein-coupled receptor kinase 5 | GRK5 | 2869 |
| 994_at | protein tyrosine phosphatase, receptor type, M | PTPRM | 5797 |
| 995_g_at | protein tyrosine phosphatase, receptor type, M | PTPRM | 5797 |
| 914_g_at | v-ets erythroblastosis virus E26 oncogene homolog (avian) | ERG | 2078 |
| 873_at | homeobox A5 | HOXA5 | 3202 |
| 767_at | myosin, heavy chain 11, smooth muscle | MYH11 | 4629 |
| 770_at | glutathione peroxidase 3 (plasma) | GPX3 | 2878 |
| 774_g_at | myosin, heavy chain 11, smooth muscle | MYH11 | 4629 |
| 758_at | prostaglandin I2 (prostacyclin) receptor (IP) | PTGIR | 5739 |
| 610_at | adrenergic, beta-2-, receptor, surface | ADRB2 | 154 |
| 560_s_at | T-cell acute lymphocytic leukemia 1 | TAL1 | 6886 |
| 538_at | CD34 molecule | CD34 | 947 |
| 481_at | SNF related kinase | SNRK | 54861 |
| 340_at | matrilin 3 | MATN3 | 4148 |
| 210_at | phospholipase C, beta 2 | PLCB2 | 5330 |
