# Supplementary material for: Yin Yang Gene Expression Ratio Signature for Lung Cancer Prognosis
Source: PLoS One. 2013 Jul 17;8(7):e68742. doi: 10.1371/journal.pone.0068742 (PMC3714286; doi:10.1371/journal.pone.0068742)
Supplement: Table S7 — Data sets and preprocesses used in this study. (DOC) [file pone.0068742.s015.doc]

**Table S7. Data sets and preprocesses used in this study**

| **Ref** | **accession** | **samples type** | **sample#** | **platform** | **preprocess** | **Normalization** |
| --- | --- | --- | --- | --- | --- | --- |
| Yanai, 2005 | GSE803 | normal tissues | 24 | HG-U95A | MAS5.0 | Quantile |
| Shyamsundar R, 2005 | GSE2193 | normal lung | 3 | 2-color DNA | Ratio | linear global |
| Crouser, 2009 | GSE16538 | Normal lung | 6 | HG-133plus2 | MAS5.0 | None |
| April c, 2009 | GSE17558 | normal & tumor | 16 | Illumina | Average | Quantile |
| Landi, 2008 | GSE10072 | normal & tumor | 107 | HG-133A | RMA | Quantile |
| Bhattacharjee, 2001 | caArray | lung cancer | 233 | HG-95A | RMA | Quantile |
| Bild,2006 | GSE3141 | Lung cancer | 58 | HG-133plus2 | MAS5.0 | median |
| Shedden, 2008 | caArray | lung cancer | 443 | HG-133A | MAS5.0 | None |
| TCGA | LUAD | adenocarcinoma | 259 | RNA-seq | RPKM | global |
