# Supplementary material for: Yin Yang Gene Expression Ratio Signature for Lung Cancer Prognosis
Source: PLoS One. 2013 Jul 17;8(7):e68742. doi: 10.1371/journal.pone.0068742 (PMC3714286; doi:10.1371/journal.pone.0068742)
Supplement: Table S8 — YMR values of the different normal tissue types. (DOC) [file pone.0068742.s016.doc]

**Table S8. YMR values of the different normal tissue types**

| **Tissue type** | **YMR** |
| --- | --- |
| boneMarrow | 1.35 |
| boneMarrow | 1.57 |
| liver | 2.46 |
| liver | 2.78 |
| heart | 0.24 |
| heart | 0.24 |
| spleen | 0.74 |
| spleen | 0.87 |
| lung | 0.37 |
| lung | 0.38 |
| kidney | 1.39 |
| kidney | 1.26 |
| skeletalMuscle | 0.22 |
| skeletalMuscle | 0.21 |
| thymus | 1.58 |
| thymus | 1.72 |
| brain | 1.17 |
| brian | 1.33 |
| spinalcord | 0.98 |
| spinalcord | 1.08 |
| prostate | 0.84 |
| prostat | 0.88 |
| pancreas | 2.07 |
| pancreas | 2.04 |

The preprocessed by MAS50.0 and quantile-normalized data was download from NCBI GEO database (GSE803). The YMRs of each sample were directly calculated from the 31 Yin gene and the 32 Yang gene arithmetic mean values.
