# Supplementary material for: Yin Yang Gene Expression Ratio Signature for Lung Cancer Prognosis
Source: PLoS One. 2013 Jul 17;8(7):e68742. doi: 10.1371/journal.pone.0068742 (PMC3714286; doi:10.1371/journal.pone.0068742)
Supplement: Table S9 — Continuous and dichotomous arithmetic YMR scores are associated with clinical outcomes. (DOC) [file pone.0068742.s017.doc]

**Table S9. Continuous and dichotomous arithmetic YMR scores are associated with clinical outcomes.**

|  |  | | | |
| --- | --- | --- | --- | --- |
| data set | **Bhattacharjee** | **Bild** | **DCC** | **RNAseq** |
| data size | 125 | 58 | 442 | 258 |
| mean YMR | 2.23 | 1.65 | 1.85 | 2.24 |
| normal sample mean YMR | 0.91 | NA | NA | 0.38 |
| continuous variable |  |  |  |  |
| log Rank-p | *0.04* | *0.50* | *0.009* | *0.007* |
| HR | 1.96 | 1.67 | 1.8 | 1.87 |
| dichotomous variable |  |  |  |  |
| YMR cutoff | >2.0 | >1.4 | >1.8 | >1.8 |
| low risk YMR | 60 | 27 | 248 | 121 |
| high risk YMR | 65 | 31 | 194 | 137 |
| log Rank-p | *0.01* | *0.02* | *0.005* | *0.007* |
| HR | 2.7 | 2.72 | 2.63 | 2.73 |
