# Supplementary material for: Yin Yang Gene Expression Ratio Signature for Lung Cancer Prognosis
Source: PLoS One. 2013 Jul 17;8(7):e68742. doi: 10.1371/journal.pone.0068742 (PMC3714286; doi:10.1371/journal.pone.0068742)
Supplement: Table S10 — Continuous and dichotomous geometric gYMR scores are associated with clinical outcomes. (DOC) [file pone.0068742.s018.doc]

**Table S10. Continuous and dichotomous geometric g**YMR scores are associated with clinical outcomes.

|  |  | | | |
| --- | --- | --- | --- | --- |
| data set | **Bhattacharjee** | **Bild** | **DCC** | **RNAseq** |
| data size | 125 | 58 | 442 | 258 |
| mean YMR | 1.55 | 1.35 | 0.96 | 1.65 |
| normal sample mean YMR | NA | NA | NA | 0.13 |
| continuous variable |  |  |  |  |
| log Rank-p | *0.46* | *0.13* | *0.0001* | *0.035* |
| HR | 1.19 | 2.02 | 1.93 | 2.04 |
| dichotomous variable |  |  |  |  |
| YMR cutoff | >1.2 | >1.0 | >0.8 | >1.2 |
| low risk | 55 | 17 | 174 | 120 |
| high risk | 70 | 41 | 268 | 138 |
| log Rank-p | *0.64* | *0.017* | *0.0001* | *0.007* |
| HR | 2.8 | 3.28 | 2.86 | 2.74 |
