# Supplementary material for: Yin Yang Gene Expression Ratio Signature for Lung Cancer Prognosis
Source: PLoS One. 2013 Jul 17;8(7):e68742. doi: 10.1371/journal.pone.0068742 (PMC3714286; doi:10.1371/journal.pone.0068742)
Supplement: Table S11 — Dropping three genes (HIST1H4J, CDC25A, and IGFBP5) on continuous and dichotomous YMR. (DOC) [file pone.0068742.s019.doc]

**Table S11. Dropping three genes (HIST1H4J, CDC25A, and IGFBP5) on** continuous and dichotomous YMR

|  |  | | | |
| --- | --- | --- | --- | --- |
| Data set | **Bhattacharjee** | **Bild** | **DCC** | **RNAseq** |
| data size | 125 | 58 | 442 | 258 |
| mean YMR | 2.43 | 1.67 | 1.995 | 1.8 |
| normal sample mean YMR | NA | NA | NA | 0.27 |
| continuous variable |  |  |  |  |
| log Rank-p | *0.04* | *0.62* | *0.008* | *0.09* |
| HR | 1.94 | 1.73 | 1.8 | 2 |
| dichotomous variable |  |  |  |  |
| YMR cutoff | >2.2 | >1.6 | >1.9 | >1.2 |
| low risk YMR | 63 | 30 | 237 | 102 |
| high risk YMR | 62 | 28 | 206 | 156 |
| log Rank-p | *0.003* | *0.03* | *0.004* | *0.007* |
| HR | 2.63 | 2.44 | 2.66 | 2.87 |
