# Supplementary material for: Yin Yang Gene Expression Ratio Signature for Lung Cancer Prognosis
Source: PLoS One. 2013 Jul 17;8(7):e68742. doi: 10.1371/journal.pone.0068742 (PMC3714286; doi:10.1371/journal.pone.0068742)
Supplement: Table S12 — Dropping three genes (HIST1H4J, CDC25A, and IGFBP5) on continuous and dichotomous gYMR. (DOC) [file pone.0068742.s020.doc]

**Table S12. Dropping three genes (HIST1H4J, CDC25A, and IGFBP5) on continuous and dichotomous gYMR**

| data set | **Bhattacharjee** | **Bild** | **DCC** | **RNAseq** |
| --- | --- | --- | --- | --- |
| data size | 125 | 58 | 442 | 258 |
| mean YMR | 1.7 | 1.36 | 1.01 | 1.65 |
| normal sample mean YMR | NA | NA | NA | 0.13 |
| continuous variable |  |  |  |  |
| log Rank-p | *0.3* | *0.13* | *2.00E-05* | *0.046* |
| HR | 2.15 | 2.0 | 1.95 | 2 |
| dichotomous variable |  |  |  |  |
| YMR cutoff | >1.6 | >1.0 | >0.9 | >1.2 |
| low risk | 65 | 17 | 209 | 119 |
| high risk | 60 | 41 | 233 | 139 |
| log Rank-p | *0.25* | *0.017* | *2.00E-05* | *0.015* |
| HR | 2.7 | 3.28 | 2.72 | 2.75 |
