# Supplementary material for: Yin Yang Gene Expression Ratio Signature for Lung Cancer Prognosis
Source: PLoS One. 2013 Jul 17;8(7):e68742. doi: 10.1371/journal.pone.0068742 (PMC3714286; doi:10.1371/journal.pone.0068742)
Supplement: Table S13 — YMR covariate and multivariate analysis using direct method [53] . (DOC) [file pone.0068742.s021.doc]

**Table S13. YMR covariate and multivariate analysis** using direct method [53]

| **Name** | **Estimate** | **Std Error** | **W (Wald Chi Square)** | **p-value (W)** | **Hazard Ratio** |
| --- | --- | --- | --- | --- | --- |
| **Covariate** |  |  |  |  |  |
| YMR | 0.39 | 0.09 | 20.32 | <1.0E-05 | 1.47 |
| **Multivariate** |  |  |  |  |  |
| YMR | 0.28 | 0.10 | 7.54 | 0.006 | 1.32 |
| chemo: yes | 0.13 | 0.18 | 0.54 | 0.463 | 1.14 |
| chemo: unknown | 0.21 | 0.25 | 0.71 | 0.401 | 1.23 |
| smoker: yes | 0.27 | 0.27 | 1.01 | 0.316 | 1.31 |
| smoker: unknown | 0.16 | 0.34 | 0.22 | 0.641 | 1.17 |
| sex: male | -0.09 | 0.15 | 0.34 | 0.557 | 0.92 |
| age: >=60 years old | -0.41 | 0.17 | 5.61 | 0.018 | 0.66 |
| stage III | 1.34 | 0.20 | 44.78 | <1.0E-10 | 3.82 |
| stage II | 0.65 | 0.18 | 13.41 | <1.0E-3 | 1.91 |
| differentiate: poor | 0.27 | 0.25 | 1.22 | 0.270 | 1.32 |
| differentiate: medium | 0.10 | 0.24 | 0.17 | 0.679 | 1.10 |

* Chemotherapy was a category variable (no chemotherapy group as reference); Smoking history was a category variable (no smoking group as reference); Sex: was a binary variable (0 for female as reference); Age was a binary variable (0 for <60 years old as reference). Tumor stage was a category variable (stage I as reference); Differentiation (well, medium, poor) was a category variable (well as reference).
