# Supplementary material for: Yin Yang Gene Expression Ratio Signature for Lung Cancer Prognosis
Source: PLoS One. 2013 Jul 17;8(7):e68742. doi: 10.1371/journal.pone.0068742 (PMC3714286; doi:10.1371/journal.pone.0068742)
Supplement: Table S14 — gYMR covariate and multivariate analysis using actuarial method [53] . (DOC) [file pone.0068742.s022.doc]

**Table S14. gYMR covariate and multivariate analysis using actuarial method [53]**

| **Name** | **coef** | **Std Error** | **Hazard Ratio (HR)** | **Lower 0.95 HR** | **Upper 0.95 HR** | **Z** | **p-value (z)** |
| --- | --- | --- | --- | --- | --- | --- | --- |
| **Covariate** |  |  |  |  |  |  |  |
| YMR | 0.64 | 0.15 | 1.9 | 1.41 | 2.56 | 4.2 | 2.00E-05 |
| **Multivariate** |  |  |  |  |  |  |  |
| YMR | 0.51 | 0.18 | 1.67 | 1.18 | 2.35 | 2.89 | 0.004 |
| chemo: yes | 0.21 | 0.18 | 1.23 | 0.87 | 1.74 | 1.18 | 0.24 |
| smoker: yes | 0.30 | 0.28 | 1.35 | 0.78 | 2.36 | 1.07 | 0.29 |
| sex: male | 0.10 | 0.17 | 1.10 | 0.79 | 1.53 | 0.57 | 0.57 |
| age: >=70 years | 0.45 | 0.18 | 1.57 | 1.11 | 2.22 | 2.56 | 0.01 |
| stage | 0.89 | 0.17 | 2.44 | 1.74 | 3.42 | 5.16 | 3.00E-07 |
| differentiate: poor | 0.09 | 0.18 | 1.09 | 0.77 | 1.54 | 0.50 | 0.62 |

* YMR was gYMR with three genes dropped. Chemo was a category variable (no chemotherapy group as reference); Smoker was a category variable (no smoking group as reference); Sex: was a binary variable (0 for female as reference); Age was a binary variable (0 for <70 years old as reference). Tumor stage was a category variable (stage I as reference); Differentiation was a category variable (well differentiation as reference).
